# Single-cell mapping reveals new markers and functions of lymphatic endothelial cells in lymph nodes

**DOI:** 10.1371/journal.pbio.3000704

**Published:** 2020-04-06

**Authors:** Noriki Fujimoto, Yuliang He, Marco D’Addio, Carlotta Tacconi, Michael Detmar, Lothar C. Dieterich

**Affiliations:** 1 Institute of Pharmaceutical Sciences, Swiss Federal Institute of Technology (ETH) Zürich, Zürich, Switzerland; 2 Department of Dermatology, Shiga University of Medical Sciences, Japan; UCSF, UNITED STATES

## Abstract

Lymph nodes (LNs) are highly organized secondary lymphoid organs that mediate adaptive immune responses to antigens delivered via afferent lymphatic vessels. Lymphatic endothelial cells (LECs) line intranodal lymphatic sinuses and organize lymph and antigen distribution. LECs also directly regulate T cells, mediating peripheral tolerance to self-antigens, and play a major role in many diseases, including cancer metastasis. However, little is known about the phenotypic and functional heterogeneity of LN LECs. Using single-cell RNA sequencing, we comprehensively defined the transcriptome of LECs in murine skin-draining LNs and identified new markers and functions of distinct LEC subpopulations. We found that LECs residing in the subcapsular sinus (SCS) have an unanticipated function in scavenging of modified low-density lipoprotein (LDL) and also identified a specific cortical LEC subtype implicated in rapid lymphocyte egress from LNs. Our data provide new, to our knowledge, insights into the diversity of LECs in murine LNs and a rich resource for future studies into the regulation of immune responses by LN LECs.

## Abstract 2

Single-cell RNA sequencing provides a comprehensive map of lymph-node–resident lymphatic endothelial cells, identifying new specific markers and functions of cell subpopulations, including the scavenging of low-density lipoprotein and mediation of rapid lymphocyte egress from lymph nodes.

## Introduction

Peripheral lymph nodes (LNs) are essential secondary lymphoid organs that mediate interactions between antigen-presenting cells and lymphocytes for the initiation of adaptive immune responses. LNs also act as filters that retain specific proteins and other biomolecules present in the afferent lymph [1]. Apart from lymphocytes, LNs comprise several stromal cell types—including fibroblastic reticular cells (FRCs), blood vascular endothelial cells, and lymphatic endothelial cells (LECs)—that are crucial for LN development and function. LECs not only provide structure to the LN sinuses that allow lymph percolation through the node but also control the access of soluble molecules and subcellular particles (including viruses) to the conduit system that guides them to dendritic cells residing in the LN cortex [2,3]. LN LECs also actively engage in a variety of immune-related processes. Under steady-state conditions, LN LECs control lymphocyte egress from LNs via generation of an S1P gradient [4] and regulate peripheral tolerance by expression and presentation of peripheral tissue self-antigens in combination with constitutive expression of programmed death ligand 1 (PD-L1) and other regulatory molecules, leading to inhibition or deletion of autoreactive CD8^+^ T cells [5,6]. Furthermore, similar to antigen-presenting cells, LN LECs have been reported to scavenge and (cross-) present exogenous antigen taken up from the lymph [7].

LNs draining inflamed tissues or tumors commonly increase in size, which is accompanied by an expansion of LECs [8,9]. LN lymphangiogenesis may be driven by soluble factors drained from the upstream tissue or by signals produced locally in the lymph node, such as B cell-derived vascular endothelial growth factor (VEGF)-A [10]. Consequently, in the case of tumor-draining LNs, lymphatic expansion can even occur before the colonization by tumor cells [11], a process that may be involved in the generation of a “pre-metastatic niche” [12]. Importantly, in pathological conditions, LN LECs not only increase in number but also adapt a distinctive molecular phenotype. Several studies have characterized transcriptional changes of bulk-isolated LN LECs in response to experimental inflammation, virus infection, and in the context of upstream tumor growth [13–15], demonstrating that LN LECs dynamically regulate a large number of genes associated with inflammatory processes, which conceivably affects their function and, subsequently, any adaptive immune responses generated in the node.

LNs are highly organized structures that host specialized immune cell types in defined anatomical compartments such as the subcapsular, cortical, and medullary regions. Therefore, it is conceivable that stromal cells parallel this zonation and display diverse phenotypes and functions, depending on their location in the node. For example, Rodda and colleagues recently identified multiple subtypes of FRCs that differed in location and gene expression [16]. Several reports have addressed the heterogeneity of LECs in murine LNs, typically focusing on selected marker genes only. One of the most prominent examples are the LECs lining the ceiling and the floor of the subcapsular sinus (SCS), which exhibit markedly different phenotypes in spite of their close physical proximity. For example, ceiling LECs (cLECs) express the atypical chemokine receptor ACKR4 and display no or only low levels of lymphatic vessel endothelial hyaluronan receptor 1 (LYVE1), whereas floor-lining LECs (fLECs) express high levels of LYVE1 but are negative for ACKR4 [17]. Further studies identified mucosal vascular addressin cell adhesion molecule 1 (MADCAM1) and integrin subunit alpha 2b (ITGA2B) as additional markers of fLECs [18–20]. The distinct molecular phenotypes of c- and fLECs likely enable them to support specific functions, such as the fLEC-specific transmigration of antigen-presenting cells [17,21] or transcytosis of antibodies [22] from the SCS into the LN cortex. However, to comprehensively define the molecular and functional heterogeneity of LN LECs, analysis at single-cell resolution is necessary.

Recently, a study by Takeda and colleagues reported a single-cell sequencing analysis of LN LECs isolated from cancer patients and described 4 subsets corresponding to SCS cLECs and fLECs, a second type of cLECs present only in the medullary region, and a single cluster of medullary and cortical LECs that were transcriptionally indistinguishable [23]. However, the sequencing depth of the transcriptional data provided in this study was relatively shallow and might have been influenced by the diseased state because of systemic responses to tumor growth. Here, we performed single-cell RNA sequencing of LECs isolated from murine inguinal LNs of completely naïve animals for unbiased identification of LEC subsets and comprehensive characterization of their phenotypes in the steady state. Our results reveal that there are at least 4 subsets of murine LN LECs with marked differences in gene expression that correspond to distinct anatomical locations within the LN. These included the cLECs and fLECs of the SCS, as well as medullary sinus (MS) LECs, similar to what has been described in humans [23]. Notably, we additionally identified a small subset of cortical sinuses that mediate rapid lymphocyte egress from the LN, and we uncovered a hitherto unknown function of LN LECs in scavenging low-density lipoproteins (LDLs).

## Results

### Identification of 4 LN LEC subtypes and gene expression signatures by single-cell sequencing

**Fig 1:**
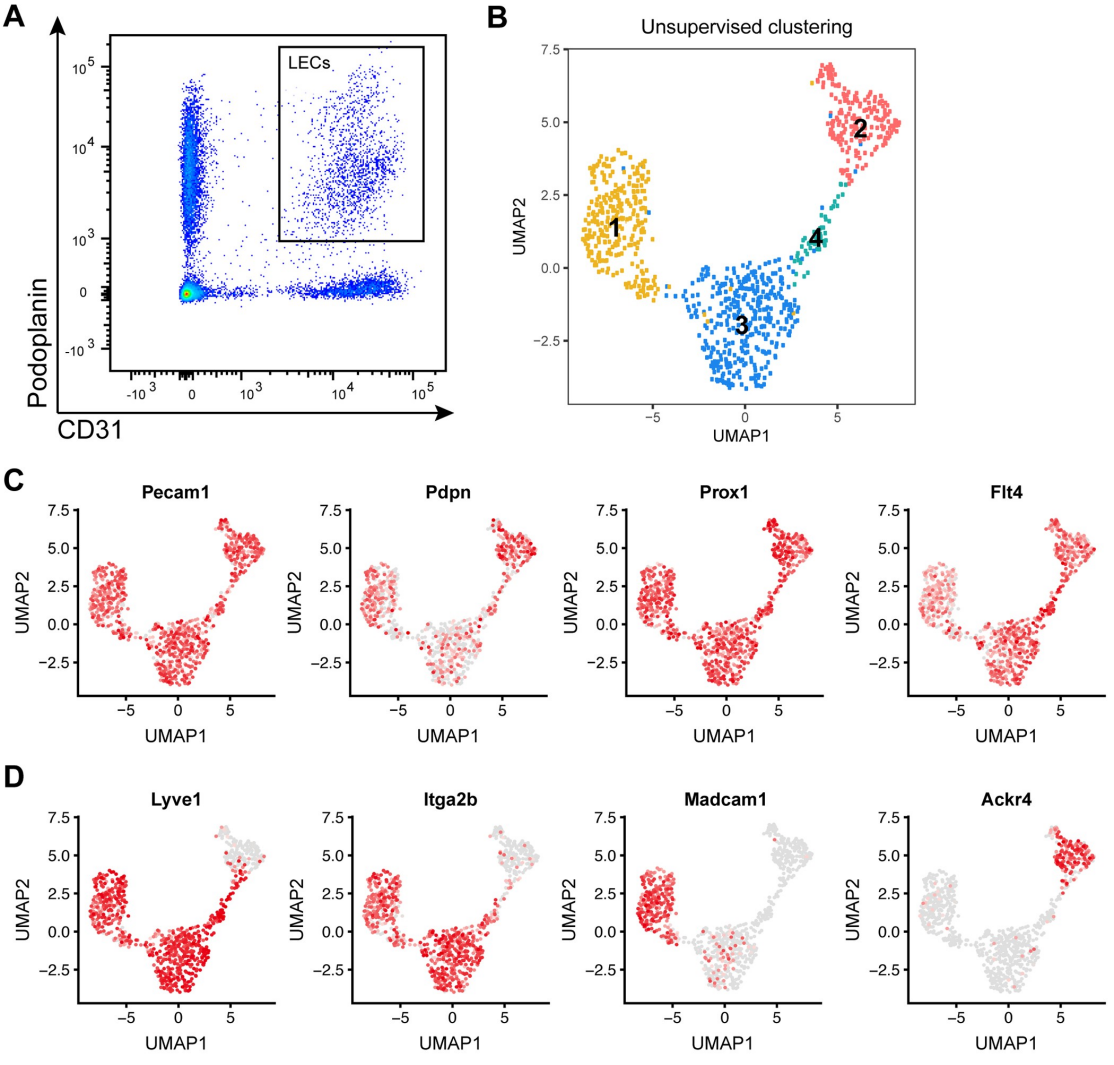
Single-cell transcriptomic analysis of LN LECs. (A) Example FACS plot of LN stromal cells (pregated as CD45^-^ living singlets) from inguinal LNs of C57Bl/6 wild-type mice. CD31^+^Pdpn^+^ LECs were isolated by single-cell sorting. (B) Unsupervised clustering of 893 LN LECs resulted in 4 distinct clusters. Each point represents an individual cell. (C, D) Expression levels of selected genes plotted using the original log-transformed counts. Gray dots indicate cells without any measurable expression; red dots coded by color intensity denote the detected expression magnitude. (C) Pecam1 (CD31) and Pdpn used as markers for FACS sorting, as well as the LEC marker genes Prox1 and Flt4 (Vegfr3), were robustly expressed in most cells. (D) While Lyve1 and Itga2b were expressed in all clusters except for cluster 2, the fLEC marker Madcam1 and the cLEC marker Ackr4 were specifically expressed in cluster 1 and cluster 2, respectively. ACKR4, atypical chemokine receptor 4; CD, cluster of differentiation; cLEC, ceiling LEC; FACS, fluorescence-activated cell sorting; fLEC, floor-lining LEC; Flt4, Fms related receptor tyrosine kinase 4; ITGA2B, integrin subunit alpha 2b; LEC, lymphatic endothelial cell; LN, lymph node; LYVE1, lymphatic vessel endothelial hyaluronan receptor 1; MADCAM1, mucosal vascular addressin cell adhesion molecule 1; Pdpn, podoplanin; Pecam1, platelet and endothelial cell adhesion molecule 1; Prox1, prospero homeobox 1; UMAP, Uniform Manifold Approximation and Projection; VEGF, vascular endothelial growth factor.

To map the heterogeneity of LN LECs, we isolated these cells (cluster of differentiation [CD]45^-^CD31^+^podoplanin [Pdpn]^+^, Fig 1A) from inguinal LNs of C57Bl/6 wild-type mice by fluorescence-activated cell sorting (FACS) and subjected them to deep RNA sequencing at single-cell resolution (*N* = 1,152 cells) using the SmartSeq2 full-length transcriptome profiling approach [43]. After quality filtering to remove cells with outlier read counts (*N* = 134) and a group of cells showing CD45 expression (*N* = 125) that was probably due to sorting impurity, 893 cells were subjected to further analysis. Unsupervised clustering suggested the existence of at least 4 LEC subtypes: the largest cluster (cluster 3) comprised 364 cells (40.8%), cluster 1 comprised 283 cells (31.7%), cluster 2 comprised 194 cells (21.7%), and the smallest cluster (cluster 4), located between cluster 2 and 3, comprised 52 cells (5.8%) (Fig 1B). All of these cells showed robust expression of the 2 markers used for FACS sorting, CD31 (platelet and endothelial cell adhesion molecule 1 [Pecam1]) and Pdpn (Fig 1C), and the LEC marker genes prospero homeobox 1 (Prox1) and Fms related receptor tyrosine kinase 4 (Flt4, also called Vegfr3) (Fig 1C), confirming their lymphatic endothelial identity. Previously, it has been reported that LECs lining the SCS show distinct marker expression depending on their location in the ceiling or the floor of the sinus. For example, cLECs are negative for LYVE1 and ITGA2B but express ACKR4, whereas fLECs express MADCAM1 [17,18,20] (S1A Fig). In agreement with this, we observed differential expression (DE) of these genes among the 4 LEC clusters. LYVE1 and ITGA2B were present in all clusters apart from cluster 2, cluster 1 specifically expressed MADCAM1, and cluster 2 specifically expressed ACKR4 (Fig 1D). This suggests that the clusters we identified based on gene expression correspond to LECs in different anatomical locations in the LN, with cluster 1 representing fLECs and cluster 2 cLECs, whereas clusters 3 and 4 most likely represent cortical and/or medullary LEC subsets.

DE analysis among the 4 clusters identified significantly (log_2_ FC ≥ 0.6; false discovery rate (FDR) < 0.01) up- or down-regulated genes in each of the clusters compared to all others (S1 Table). Interestingly, gene ontology (GO) analysis of these DE genes suggested “opposing” phenotypes of cluster 1 LECs (fLECs) and cluster 2 LECs (cLECs), with an enrichment of inflammation-associated genes and a de-enrichment of angiogenesis-associated transcripts in cluster 1 and, vice versa, an enrichment of angiogenesis-associated and a de-enrichment of inflammation-associated genes in cluster 2 (S2 Table). Cluster 4 LECs also showed an enrichment of angiogenesis-related transcripts, whereas cluster 3 was characterized by metabolism- and oxidation-related terms (S3 Table).

### Molecular characterization of LECs in the SCS floor

**Fig 2:**
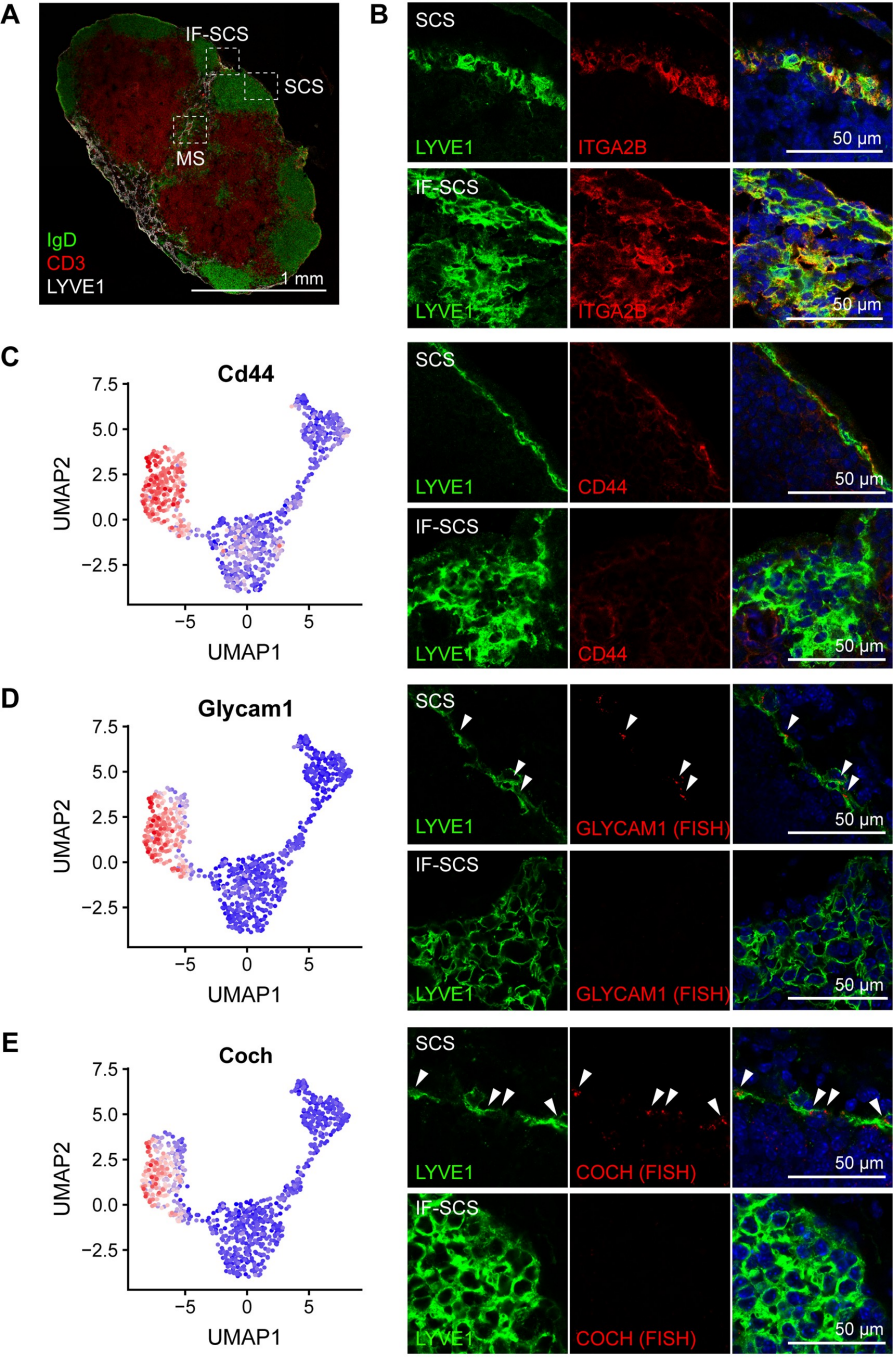
Molecular characterization of LECs in the SCS floor. (A) Immunofluorescence staining of a whole inguinal LN cross-section for IgD (green), CD3 (red), and LYVE1 (white). The dotted squares indicate 3 regions that were used for further analysis: the SCS (SCS associated with B cell follicles), the IF-SCS (SCS and large IF sinus tracts entering the node), and the MS within the node. (B) Immunofluorescence costaining for LYVE1 (green) and ITGA2B (red) in the SCS (upper panels) and the IF-SCS region (lower panels) showed expression of ITGA2B and LYVE1 by LECs in both the SCS floor and the IF-SCS region. (C–E) Gene expression patterns (left panels; denoted as high expression level in red and low in blue, using the corrected expression values) and protein/transcript location of new fLEC (cluster 1) markers. CD44 (C, detected by immunofluorescence staining), Glycam1 (D, detected by RNA FISH), and Coch (E, detected by RNA FISH) were specifically expressed in fLECs in the SCS region (upper right panels), but not in the IF-SCS region (lower right panels). White arrowheads indicate RNA FISH signals; LYVE1 stained in green. CD, cluster of differentiation; cLEC, ceiling LEC; Coch, cochlin; FISH, fluorescence in situ hybridization; fLEC, floor-lining LEC; Glycam1, glycosylation-dependent cell adhesion molecule 1; IF, interfollicular; IgD, immunoglobulin D; ITGA2B, integrin subunit alpha 2b; LEC, lymphatic endothelial cell; LN, lymph node; LYVE1, lymphatic vessel endothelial hyaluronan receptor 1; MS, medullary sinus; SCS, subcapsular sinus; UMAP, Uniform Manifold Approximation and Projection.

To confirm the identity and the anatomical location of the LEC clusters, we performed immunofluorescence staining and RNA detection in situ in inguinal LN tissue sections. To evaluate the distribution of marker expression, we selected 3 regions within the LN: (1) the SCS associated with B cell follicles, (2) the SCS and large interfollicular sinus tracts entering the nodes between the follicles (denoted as IF-SCS), and (3) the MSs within the node (Fig 2A). To validate that cluster 1 LECs were accurately assigned to the fLECs, we first analyzed the expression of the known fLEC-expressed genes ITGA2B and LYVE1. As expected, these 2 markers were clearly detectable in fLECs in the SCS and more broadly in the IF-SCS regions (Fig 2B; S1B Fig). Similarly, ACKR4^+^ cLECs were detectable in both the SCS and the IF-SCS region (S1C and S1D Fig).

Next, we chose several new markers significantly up-regulated in cluster 1/fLECs (S1 Table) for validation. CD44, a homologue of LYVE1 [24], was specifically expressed in cluster 1 LECs. In agreement with this, immunofluorescence staining of CD44 was found in LYVE1^+^ fLECs in the SCS region. Interestingly however, it was largely absent from the IF-SCS region (Fig 2C), suggesting that the cluster 1/fLEC subset is only present in the SCS right above B cell follicles, whereas most of the LECs in IF-SCS regions display a different phenotype that instead corresponds to cluster 3. In line with this, Glycam1 and cochlin (Coch), 2 additional cluster-1–specific transcripts, could also be detected in fLECs in the SCS region by in situ RNA hybridization but were absent from the IF-SCS region (Fig 2D and 2E).

### Identification of new markers and functions of LECs in the SCS ceiling

**Fig 3:**
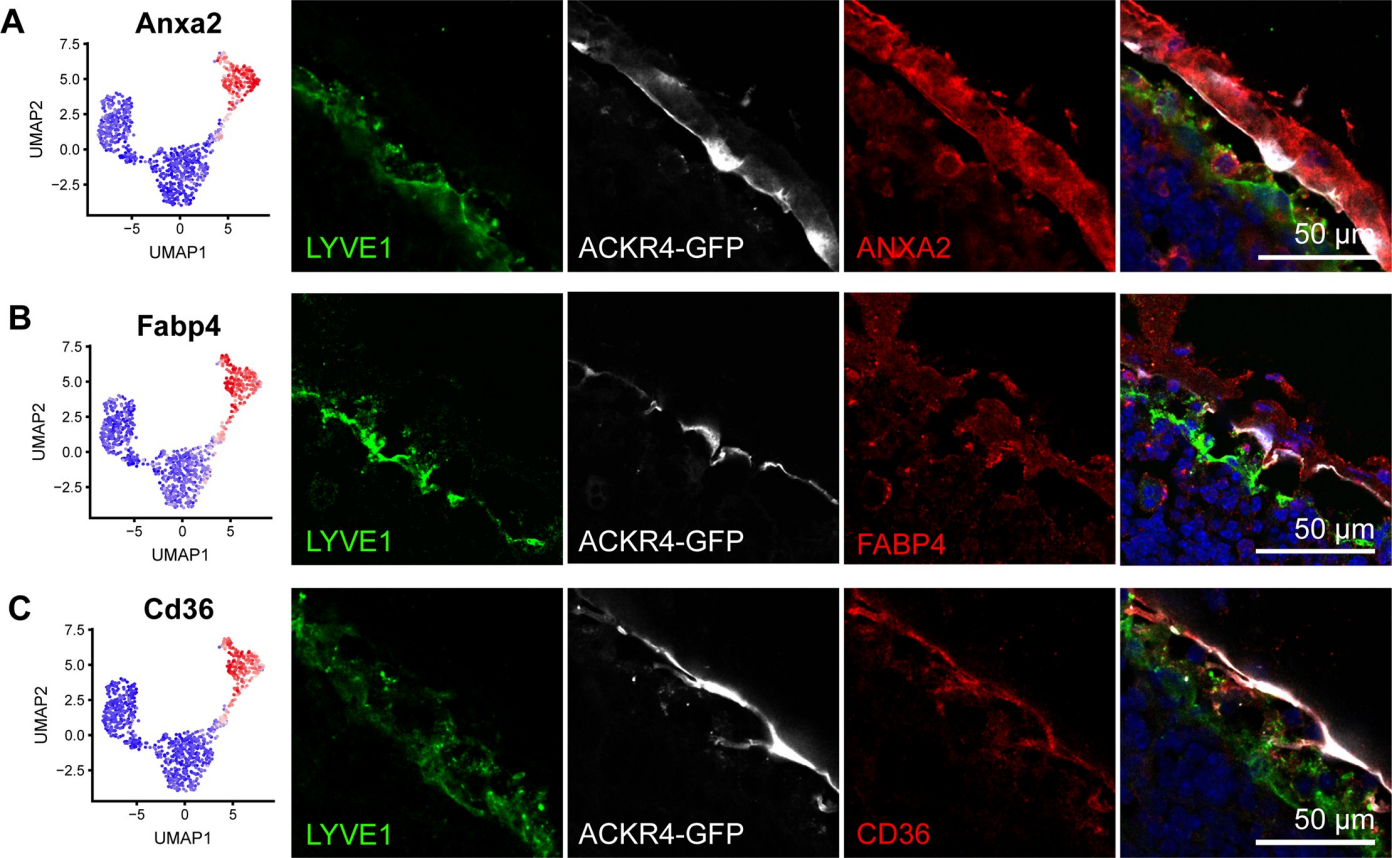
Molecular characterization of LECs in the SCS ceiling with immunofluorescence staining. (A–C) Expression of new cLEC/cluster 2 marker genes ANXA2 (A), FABP4 (B), and CD36 (C) by RNA sequencing (left panels) and immunofluorescence staining (right panels) in Ackr4-GFP reporter mice. GFP (white) and immunofluorescence costaining for LYVE1 (green) served as markers for cLECs and fLECs, respectively. ACKR4, atypical chemokine receptor 4; ANXA2, annexin A2; CD, cluster of differentiation; cLEC, ceiling LEC; FABP4, fatty acid binding protein 4; fLEC, floor-lining LEC; GFP, green fluorescent protein; LEC, lymphatic endothelial cell; LYVE1, lymphatic vessel endothelial hyaluronan receptor 1; SCS, subcapsular sinus; UMAP, Uniform Manifold Approximation and Projection.

**Fig 4:**
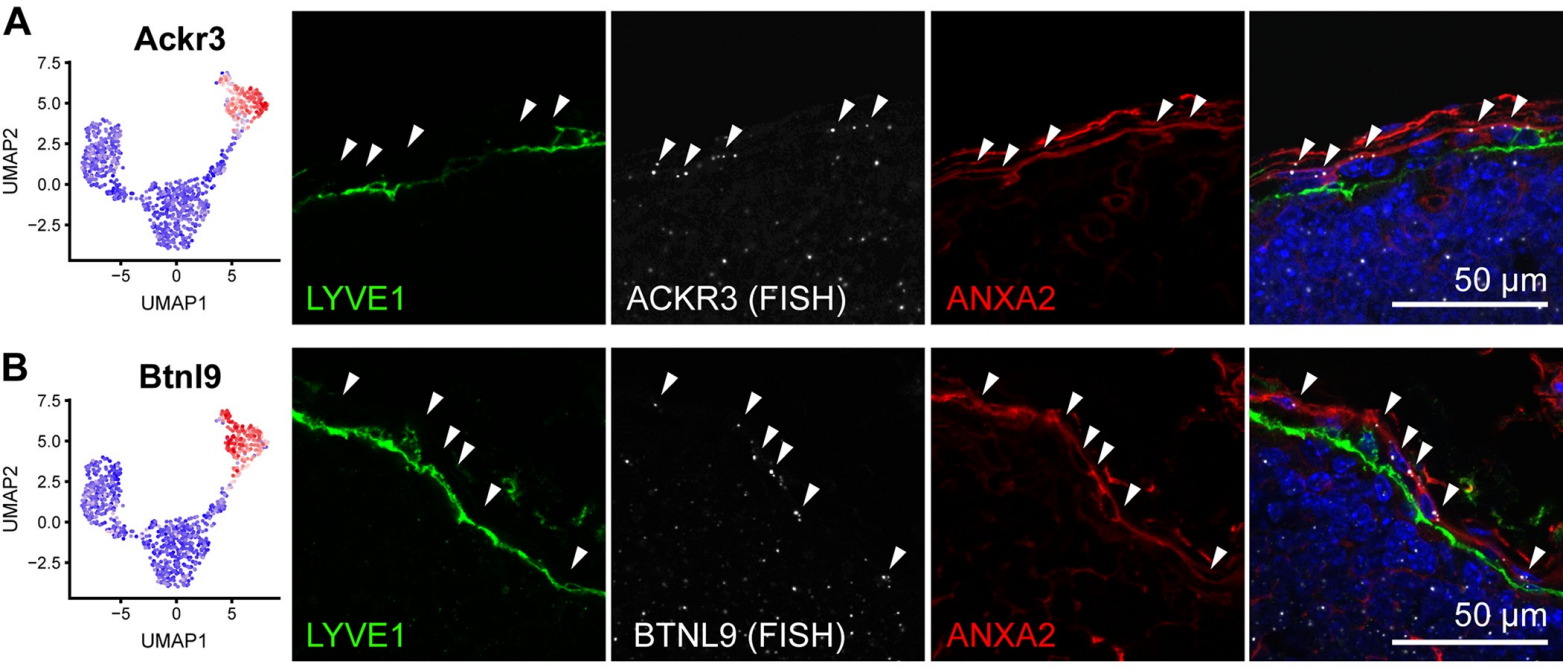
Molecular characterization of LECs in the SCS ceiling with RNA FISH. (A-B) Expression of new cLEC/cluster 2 marker genes Ackr3 (A) and Btnl9 (B) by RNA sequencing (left panels) and RNA FISH (right panels). As GFP fluorescence is lost during tissue processing for RNA FISH, immunofluorescence staining for ANXA2 (red) and LYVE1 (green) served as markers for cLECs and fLECs, respectively. Arrows point to cLECs expressing Ackr3 and Btnl9 transcripts (white). ACKR3, atypical chemokine receptor 3; ANXA2, annexin A2; Btnl9, butyrophilin like 9; cLEC, ceiling LEC; FISH, fluorescence in situ hybridization; fLEC, floor-lining LEC; LEC, lymphatic endothelial cell; LYVE1, lymphatic vessel endothelial hyaluronan receptor 1; SCS, subcapsular sinus; UMAP, Uniform Manifold Approximation and Projection.

Since ACKR4 is a well-established marker of cLECs [17], we used Ackr4-green fluorescent protein (GFP) reporter mice to characterize the expression of potential new cLEC marker genes. Of note, cLECs (cluster 2 LECs) were the most distinguishable LN LEC subset in our data set, with a total of 220 up- and 149 down-regulated genes in this cluster compared to all the other clusters (S1 Table). We selected several of these genes that were suitable for immunofluorescence staining and investigated their expression patterns in inguinal LNs. Annexin A2 (ANXA2) was highly expressed in cluster 2/cLECs in our data set, and we correspondingly found it located in the SCS ceiling and the capsule, partially overlapping with ACKR4-driven GFP expression (Fig 3A). Interestingly, ANXA2 also stained afferent lymphatic vessels merging with the SCS, demonstrating a phenotypic similarity between cLECs and afferent lymphatic collectors (S2A Fig). Similarly, fatty acid binding protein 4 (FABP4), CD36, fibronectin leucine rich transmembrane protein 2 (FLRT2), and biglycan (BGN) were also confined to the SCS ceiling (Fig 3B and 3C; S2B and S2C Fig). Our sequencing data furthermore indicated that cLECs specifically express another atypical chemokine receptor, Ackr3, as well as butyrophilin like 9 (Btnl9), which is related to the costimulatory B7 gene [25]. Owing to the shortage of commercially available antibodies, we localized the corresponding transcripts using RNA in situ hybridization. Expression of both Ackr3 and Btnl9 mRNA was detectable in various regions and cell types of inguinal LNs but, within the lymphatic endothelium, was confined to cLECs (Fig 4A and 4B).

**Fig 5:**
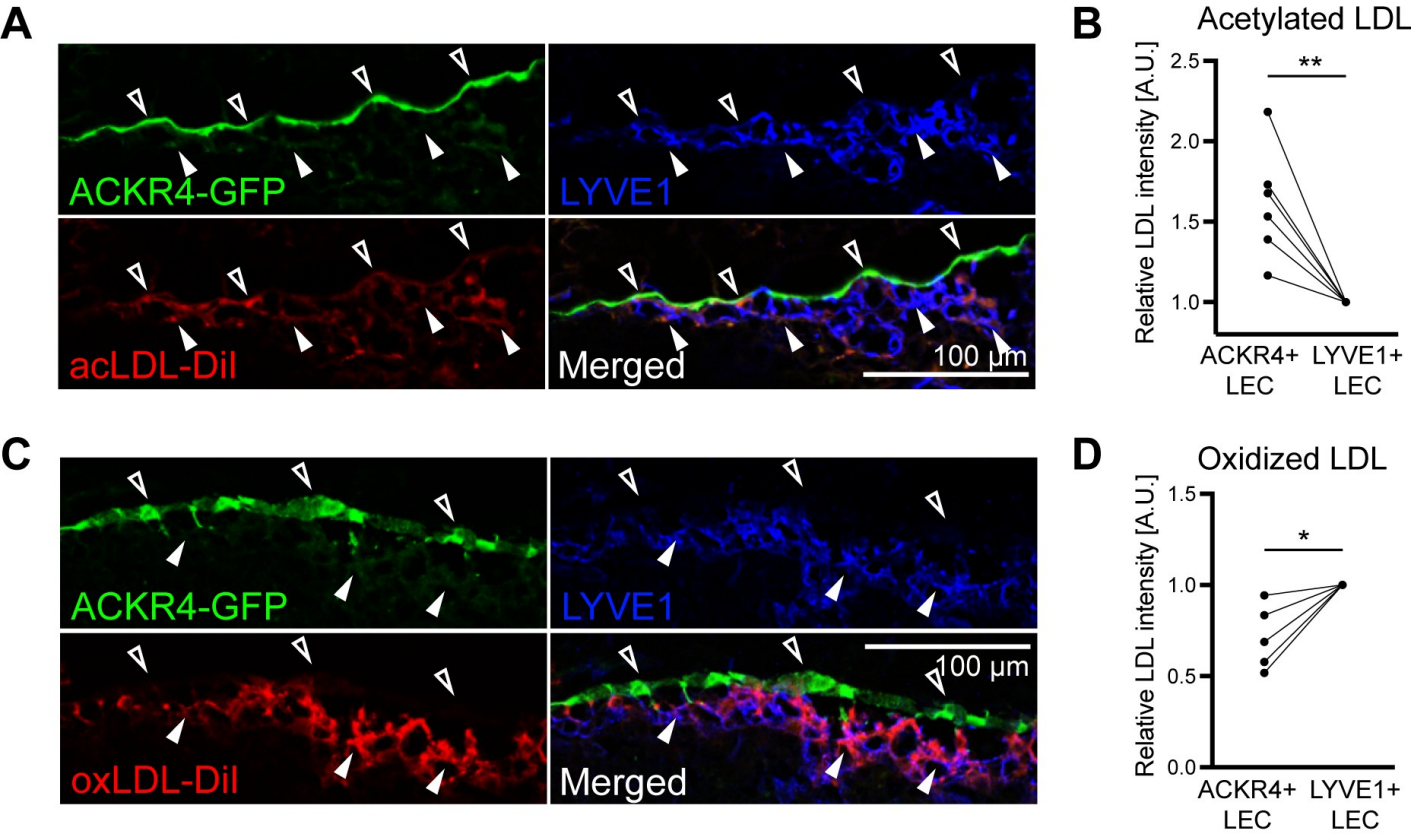
Differential LDL uptake by cLECs. In vivo LDL tracing after intradermal injection of Dil-labeled acetylated or oxidized LDL near the base of the tail of Ackr4-GFP reporter mice. Draining inguinal LNs were collected 1 h later. (A, B) Representative images (A) and quantification (B) of acetylated LDL (red) accumulation in ACKR4+ and LYVE1+ LECs. Each line represents one mouse (*n* = 6). (C–D) Representative images (C) and quantification (D) of oxidized LDL (red) accumulation in ACKR4+ and LYVE1+ LECs. Empty arrowheads indicate cLECs, and filled arrowheads indicated fLECs. For quantification, signal intensities were normalized to the level of ACKR4- LYVE1+ LECs (*n* = 5). **p < 0.01; *p < 0.05 (paired *t* test). For raw quantitative data, please refer to S1 Data. ACKR4, atypical chemokine receptor 4; A.U., arbitrary unit; cLEC, ceiling LEC; Dil, 1,1′-dioctadecyl-3,3,3′,3′-tetramethylindocarbocyanine perchlorate; fLEC, floor-lining LEC; GFP, green fluorescent protein; LDL, low-density lipoprotein; LEC, lymphatic endothelial cell; LN, lymph node; LYVE1, lymphatic vessel endothelial hyaluronan receptor 1.

Surprisingly, DE analysis identified genes known to be involved in the uptake of modified LDLs [26]. For example, CD36 was specifically expressed in cLECs (Fig 3), whereas macrophage scavenger receptor 1 (Msr1) and Fc fragment of IgG receptor IIb (Fcgr2b) were excluded from cLECs but highly expressed in most other LN LECs (S1 Table). This prompted us to evaluate whether cLECs would have a distinct capacity to take up modified LDL from the lymph. To this end, we injected Ackr4-GFP mice intradermally with fluorescently labeled acetylated or oxidized LDL near the base of the tail and collected the draining inguinal LNs 1 h later. Histological analysis revealed striking differences in LDL distribution in the LN LECs. Acetylated LDL partly overlapped with ACKR4^+^ cLECs, indicating selective uptake by this LEC subset (Fig 5A and 5B), whereas oxidized LDL was instead taken up by LYVE1^+^ LECs in the SCS floor and in cortical regions (Fig 5C and 5D). These data reveal a novel, to our knowledge, function of LECs in scavenging of LDLs from the lymph and furthermore suggest that cLECs and other LN LECs have a distinct capacity to take up differentially modified LDLs.

### LECs in medullary and IF sinuses are phenotypically similar

**Fig 6:**
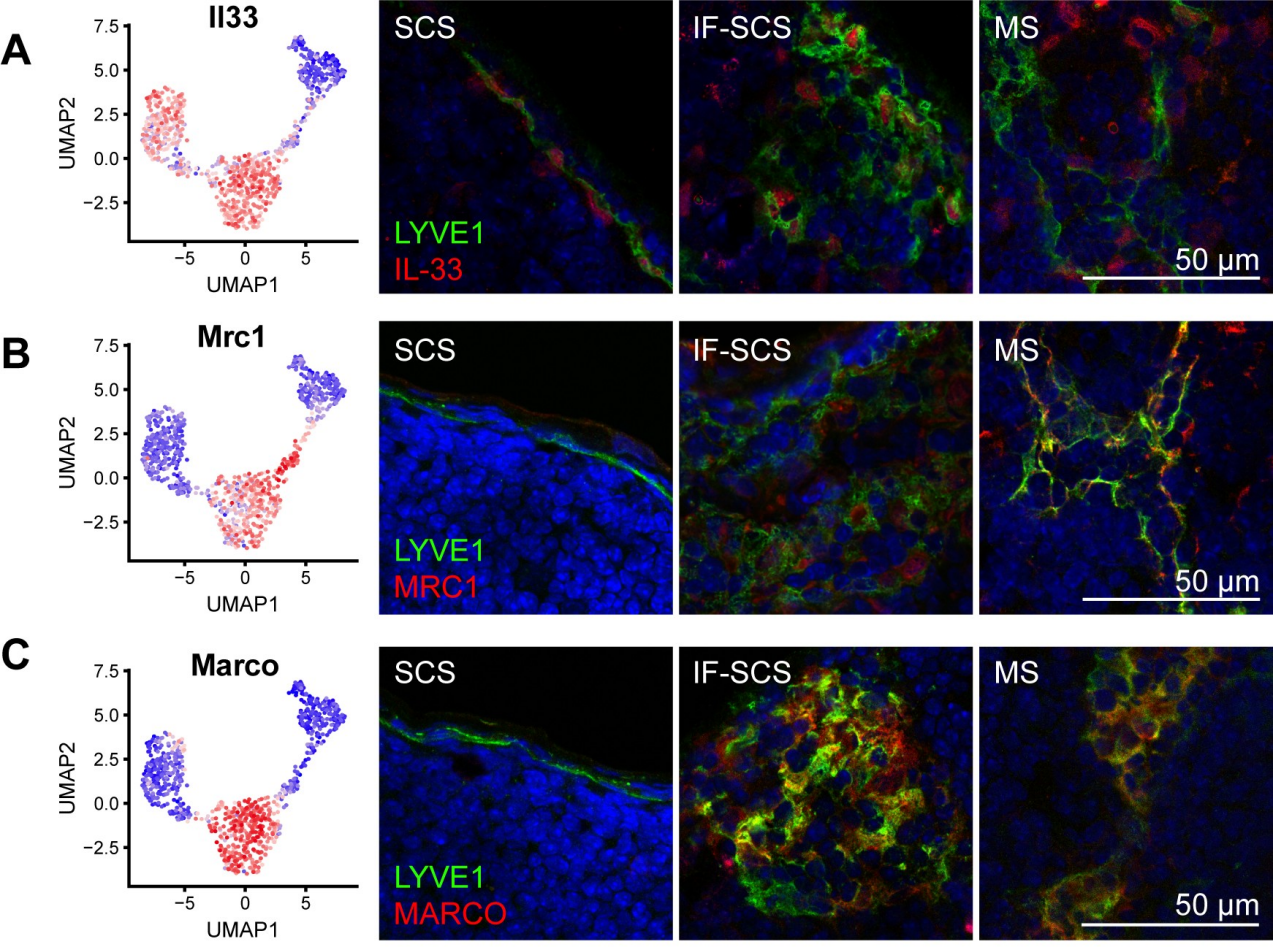
Molecular characterization of MS LECs. (A–C) Expression of MS LEC/cluster 3 marker genes IL33 (A), MRC1 (B), and MARCO (C) by RNA sequencing (left panels) and immunofluorescence staining (right panels, red) in combination with LYVE1 (green). IL33 (A) was expressed by fLECs and MS LECs and showed nuclear LEC staining in the SCS, IF-SCS, and MS regions, whereas MRC1 (B) and MARCO (C) were excluded from fLECs and congruously showed no staining in the SCS region. fLEC, floor-lining LEC; IF, interfollicular; IL33, interleukin 33; LEC, lymphatic endothelial cell; LYVE1, lymphatic vessel endothelial hyaluronan receptor 1; MARCO, macrophage receptor with collagenous structure; MRC1, mannose receptor C-type 1; MS, medullary sinus; SCS, subcapsular sinus; UMAP, Uniform Manifold Approximation and Projection.

Cluster 3 was the most abundant LEC subset in our sequencing data set and therefore likely represented cells lining the medullary and/or cortical sinuses. To test this hypothesis, we chose several marker genes expressed by cluster 3, namely interleukin 33 (IL33) (which was also expressed in fLECs) as well as mannose receptor C-type 1 (MRC1) and macrophage receptor with collagenous structure (MARCO), 2 genes typically associated with macrophages but that have previously been shown to be expressed by human LN LECs [27]. Immunofluorescence staining confirmed expression of IL33 in fLECs in the SCS and most LECs in the IF-SCS and MS regions (Fig 6A). Conversely, MRC1 and MARCO were absent from fLECs as expected but were expressed by medullary LECs in both the IF-SCS and MS regions (Fig 6B and 6C). These data indicate that cluster 3 LECs represent large IF and MSs, which consequently appear to have a very similar phenotype.

### New LEC-subset–specific markers are largely conserved among LNs from various anatomical locations and allow subset discrimination by flow cytometry

Next, we sought to investigate whether the expression pattern of the new markers for cLECs, fLECs, and medullary LECs we identified in inguinal LNs would be similar in LNs residing at other anatomical locations and therefore draining organs other than the skin, such as mandibular (draining facial regions as well as the brain [28]), iliac (draining predominantly the lower gastrointestinal tract), and mesenteric LNs (draining the upper gastrointestinal tract). Immunofluorescence staining of several selected markers (CD44, ANXA2, CD36, MRC1) revealed conserved expression patterns in these nodes, with the sole exception of CD44, which was not expressed in mesenteric fLECs (S3 Fig). Additionally, we found that some of these markers are also suitable to discriminate between the major LN LEC subsets by flow cytometry. Using inguinal LNs from Ackr4-GFP mice, a combination of ITGA2B, CD44, and MRC1 allowed us to distinguish between cLECs (GFP^+^, MRC1^-^, ITGA2B^-^, CD44^lo^), fLECs (GFP^-^, MRC1^-^, ITGA2B^+^, CD44^+^), and medullary LECs (GFP^-^, MRC1^+^, ITGA2B^+/lo^, CD44^-^) (S4 Fig).

### A unique subset of cortical and MSs serves as lymphocyte egress structures

**Fig 7:**
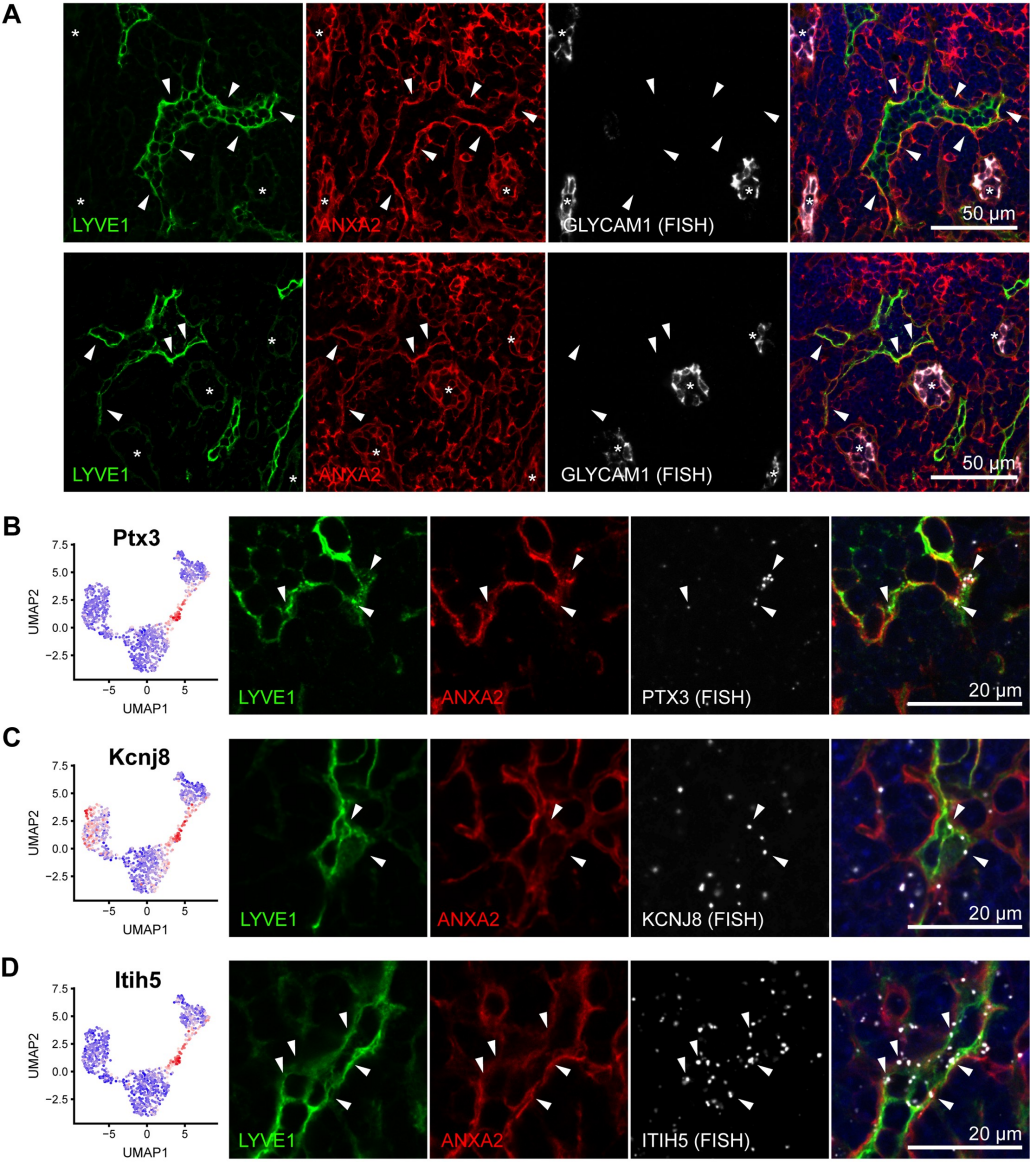
A unique subset of cortical and MSs. (A) Representative images of immunofluorescence staining for LYVE1 (green) and ANXA2 (red), in combination with RNA FISH to detect Glycam1 (white). ANXA2+ lymphatic sinuses are present in the cortex, close to the medulla (indicated by white arrowheads), and are often in close proximity to Glycam1+ HEVs (indicated by asterisks). (B–D) Expression of the cluster 4 LEC marker genes Ptx3 (B), Kcnj8 (C), and Itih5 (D) by RNA sequencing (left panels) and RNA FISH (right panels). Immunofluorescence staining for ANXA2 (red) and LYVE1 (green) was used to highlight cluster 4 sinuses. White arrowheads point to LECs expressing Ptx3, Kcnj8, and Itih5 transcripts (white). ANXA2, annexin A2; FISH, fluorescence in situ hybridization; Glycam1, glycosylation-dependent cell adhesion molecule 1; HEV, high endothelial venule; Itih5, inter-alpha-trypsin inhibitor heavy chain 5; Kcnj8, potassium inwardly rectifying channel subfamily J member 8; LYVE1, lymphatic vessel endothelial hyaluronan receptor 1; MS, medullary sinus; Ptx3, pentraxin 3; UMAP, Uniform Manifold Approximation and Projection.

The smallest LN LEC subset, cluster 4, shared the expression of many genes with medullary LECs (cluster 3) and with cLECs (cluster 2). For example, cluster 4 LECs expressed both LYVE1 as well as intermediate levels of the otherwise cLEC-restricted marker ANXA2 (Figs 1D and 3A). Interestingly, immunofluorescence staining of these 2 markers identified a subset of lymphatic sinuses located in the (para-) cortex, close to the medulla of inguinal LNs, frequently in proximity to high endothelial venules (HEVs) strongly positive for Glycam1 [29] (Fig 7A). Furthermore, they were surrounded and filled by B and T lymphocytes but rarely by F4/80^+^ or CD169^+^ macrophages, further distinguishing them from medullary sinuses (S5A–S5D Fig). We also confirmed that those structures were indeed lymphatic sinuses by staining for Prox1 and that they expressed MRC1 but were negative for MARCO (S5E–S5G Fig), as suggested by the RNA-sequencing data (Fig 6B and 6C). To further confirm that cluster 4 LECs indeed correspond to those sinuses, we selected several transcripts specific for this cluster, namely pentraxin 3 (Ptx3), potassium inwardly rectifying channel subfamily J member 8 (Kcnj8), and inter-alpha-trypsin inhibitor heavy chain 5 (Itih5), and mapped them by in situ RNA hybridization. In all cases, expression outside of lymphatic sinuses could be detected, which might be derived from other LN stromal cells or immune cells. However, within LYVE1^+^ lymphatic structures, these transcripts were only detectable in ANXA2^+^ sinuses (Fig 7B–7D). Taken together, this demonstrates that the cluster 4 LECs identified by single-cell RNA sequencing correspond to a unique subset of lymphatic sinuses in the cortex of mouse inguinal LNs.

**Fig 8:**
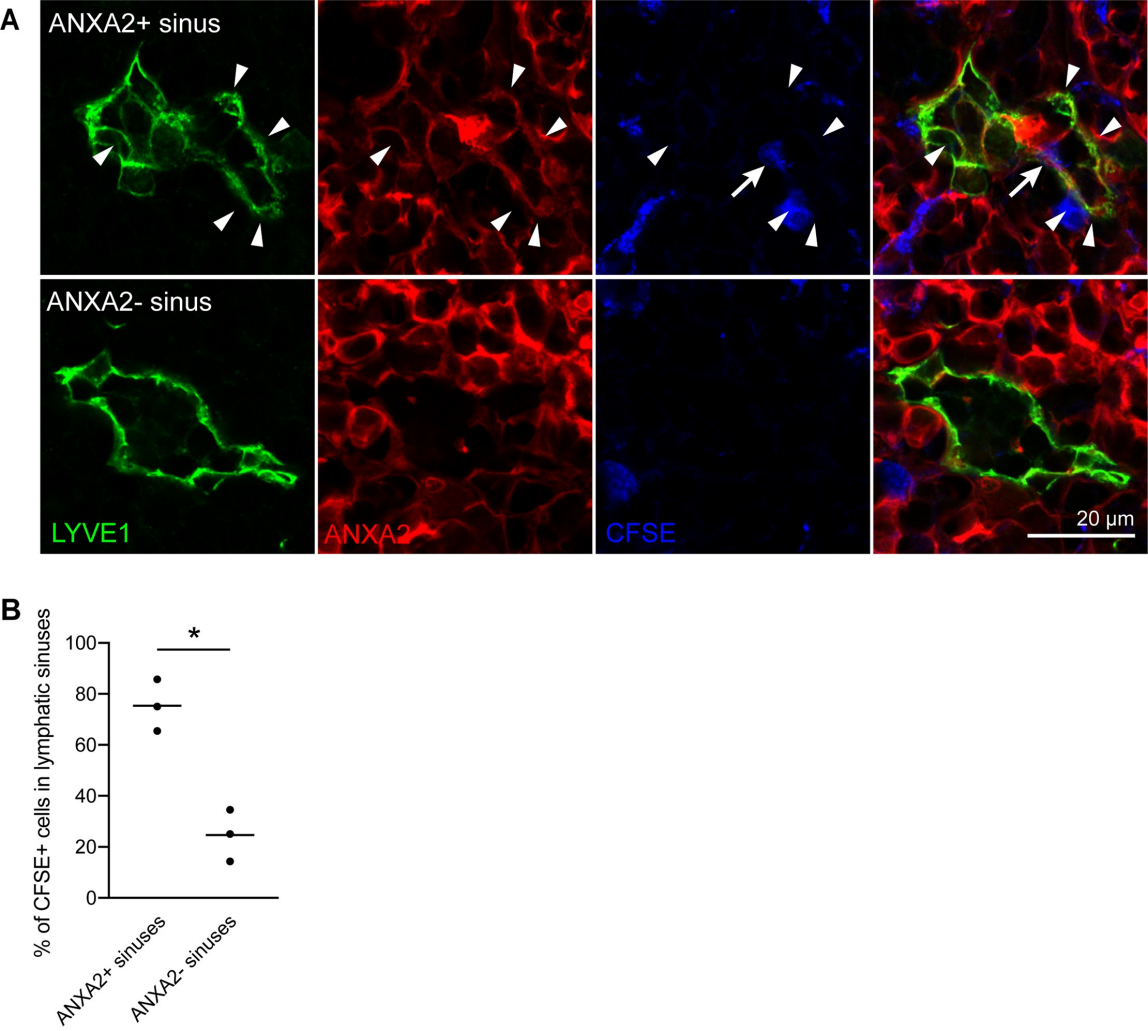
Lymphocytes egress from LNs via cluster 4 sinuses. CFSE-labeled splenocytes were infused via the tail vein, and inguinal LNs were collected 30 min later. (A) Representative images of an ANXA2+ sinus (top) and an ANXA2- sinus (bottom), stained for LYVE1 (green) and ANXA2 (red). Arrowheads indicate ANXA2+ LECs. CFSE-labeled splenocytes (blue) eventually entered lymphatic sinuses (arrow). (B) Quantification of CFSE+ splenocytes that entered ANXA2+ cluster 4 sinuses or other (ANXA2-) lymphatic sinuses 30 min after infusion, expressed as percentage of splenocytes that entered any kind of lymphatic sinus. CFSE-labeled cells were more frequently observed in ANXA2+ cluster 4 sinuses than in ANXA2- medullary sinuses. Each symbol represents one mouse (*n* = 3). *p < 0.05 (paired *t* test). For raw quantitative data, please refer to S1 Data. ANXA2, annexin A2; CFSE, carboxy-fluorescein diacetate succinimidyl ester; LEC, lymphatic endothelial cell; LN, lymph node; LYVE1, lymphatic vessel endothelial hyaluronan receptor 1.

Previously, a subset of blind-ended sinuses has been described in the cortex of rat and mouse LNs that connect to MSs and may act as rapid egress structures for lymphocytes entering the LN through adjacent HEVs [30–32]. Because of the close proximity of some cluster 4 sinuses to HEVs (Fig 7A), we hypothesized that they might be identical to those egress structures. To further investigate this hypothesis, we intravenously injected carboxy-fluorescein diacetate succinimidyl ester (CFSE)-labeled splenocytes into syngeneic recipient mice and analyzed their location in inguinal LNs after 30 min. In line with previously published data [30], we found that many of the labeled cells had reached the LN parenchyma at this time point, and some of them (approximately 5%) began to enter lymphatic sinuses. Using ANXA2 as a marker for cluster 4 sinuses as compared to MSs, we then quantified the percentage of infused splenocytes in each of the 2 sinus subtypes. Strikingly, infused cells were significantly more prevalent in ANXA2^+^ cortical sinuses than in ANXA2^-^ MSs (Fig 8A and 8B), although cortical sinuses were generally less frequent than MSs. Together, these data further indicate that the cluster 4 LECs indeed represent the previously described lymphocyte egress structures in the LN cortex.

### Murine and human LN LEC subsets are partially conserved

**Fig 9:**
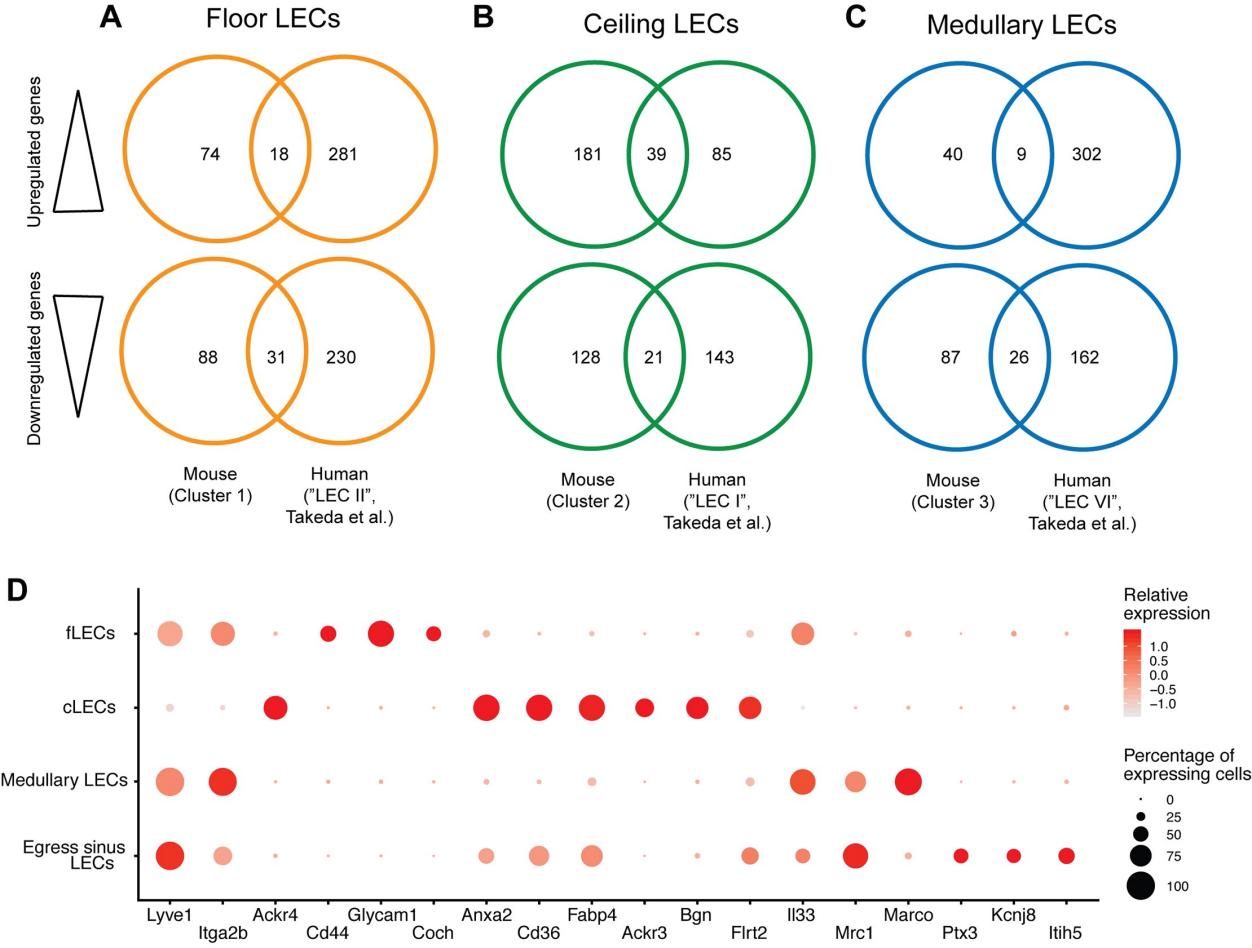
Overview of murine LN LEC subsets and comparison to human LN LECs. (A–C) Gene-level comparison between mouse and human [23] fLECs (A), cLECs (B), and medullary LECs (C), based on up-regulated (top row) and down-regulated (bottom) genes in each cluster compared to all other LECs. Venn diagrams display the number of differentially expressed genes that are shared or different between the 2 data sets. (D) Expression of the selected markers (x-axis) signifying individual LN LEC subsets (y-axis). Dot size denotes the proportion of cells with detectable expression. Color intensity indicates the relative mean expression level of the corresponding gene, using the original log-transformed counts. For raw quantitative data, please refer to S1 Data. ACKR4, atypical chemokine receptor 4; ANXA2, annexin A2; Bgn, biglycan; CD, cluster of differentiation; cLEC, ceiling LEC; Coch, cochlin; FABP4, fatty acid binding protein 4; fLEC, floor-lining LEC; Flrt2, fibronectin leucine-rich transmembrane protein 2; Glycam1, glycosylation-dependent cell adhesion molecule 1; IL33, interleukin 33; ITGA2B, integrin subunit alpha 2b; Itih5, inter-alpha-trypsin inhibitor heavy chain 5; Kcnj8, potassium inwardly rectifying channel subfamily J member 8; LEC, lymphatic endothelial cell; LN, lymph node; LYVE1, lymphatic vessel endothelial hyaluronan receptor 1; MARCO, macrophage receptor with collagenous structure; MRC1, mannose receptor C-type 1; Ptx3, pentraxin 3.

Recently, Takeda and colleagues performed a single-cell RNA-sequencing–based characterization of human LECs isolated from cervical and axillary LNs of tumor patients [23]. Similar to our study, the authors found 4 subsets of LN LECs within the nodes, including fLECs, cLECs, and medullary LECs, suggesting that these major LN LEC subtypes are conserved between species. To further characterize the phenotypic conservation of these subsets across species, we compared human [23] and mouse fLECs, cLECs, and medullary LECs in terms of up- and down-regulated genes in those LEC subtypes compared to all other LECs in the corresponding data sets. This analysis revealed certain transcriptional similarities, which were most evident in cLECs (Fig 9A–9C; S3 Table). The majority of the up- and down-regulated genes, however, were different between the corresponding murine and human LN LEC subsets.

## Discussion

To unravel the phenotypic and functional heterogeneity of LN LECs, we performed single-cell RNA sequencing coupled with unsupervised clustering and identified at least 4 LEC subtypes that differed considerably in their transcriptome. Several previously described markers of LN LEC subsets residing in specific anatomical locations, such as ACKR4, LYVE1, and ITGA2B [17,20], were differentially expressed among these clusters, confirming the validity of our sequencing data and clustering approach. Most notably, the identification of new markers (Fig 9D) will enable the isolation of individual LN LEC subsets to study their transcriptomic alterations under pathological conditions in much greater details. The large number of differentially expressed genes furthermore implies that there are functional differences between LECs residing in different areas of the LN, which is most clearly seen in case of cLECs and fLECs lining the SCS. The fLECs function as a receptive surface for antigen-presenting cells entering the SCS with the afferent lymph [17,21]. In agreement with this, we found a significant enrichment of transcripts associated with inflammatory processes, including adhesion molecules such as CD44 and Glycam1, chemokines, and the innate-immunity–related Coch [33] in these cells (S1 Table, S2 Table). cLECs, on the other hand, expressed several matrix proteins that are likely involved in giving structural support to the LN and in providing a barrier towards the surrounding tissue. We also noted specific expression of platelet-derived growth factors (PDGFs) in cLECs (S1 Table), which probably mediate recruitment of perivascular supportive cells to the LN capsule [18].

Unexpectedly, we observed several proteins involved in the cellular uptake of modified LDLs [26] to be differentially expressed between cLECs and fLECs, namely CD36 (highly expressed in cLECs), MSR1, and FCGR2B (both excluded from cLECs) (Fig 3; S1 Table). While human lymph is basically devoid of very low-density lipoproteins, it does contain LDLs [34]. In addition, the concentration of apolipoprotein B (ApoB) protein is strongly reduced in efferent compared to afferent lymph in rats [1], suggesting that the lymphatic system may be involved in cholesterol transport and that LNs can actively remove LDLs from the lymph. Our data using fluorescently labeled modified LDLs suggest that LN LECs are at least partly responsible for the scavenging of lymphatic LDL. The difference between acetylated LDL, which was selectively taken up by cLECs, and oxidized LDL, which was selectively taken up by fLECs and cortical LECs, is probably due to differences in receptor affinities. It has been reported that MSR1 and FCGR2B require a high degree of LDL oxidation for efficient binding [35], which is typically the case for commercially available oxidized LDL preparations, including the one used in the present study. Conversely, CD36 has a high affinity even for lowly oxidized LDLs [35], which may be reflected by the behavior of acetylated LDL in our assay. Clearly, further investigations are needed to examine the physiological significance of the capacity and selectivity of LDL uptake by LN LEC subtypes.

In 1985, Yechun He reported a subset of blind-ended lymphatic structures in proximity to HEVs in the inner cortex of rat mesenteric LNs, which he named the “lymphatic labyrinth” [31]. These structures were directly connected to MSs and supposedly act as an immediate egress portal for naïve lymphocytes entering the LN via the HEVs [30–32]. Here, we have identified a transcriptionally distinct LN LEC subset that most likely represents the “lymphatic labyrinth” in mouse inguinal LNs. The LECs in this subset (cluster 4) shared high expression of several genes with either cLECs or medullary LECs but also expressed several unique markers, including PTX3, ITIH5, and KCNJ8, which we confirmed using in situ RNA hybridization. Lymphocyte egress from LNs depends on S1P gradients, which are established by LECs via expression of S1P kinases (sphingosine kinase [SPHK]1, SPHK2, [36]), S1P lyase (SGPL1, [37]), and the sphingolipid transporter SPNS2 [38]. In line with this, cluster 4 LECs expressed significantly more SPNS2 than other LN LECs (S1 Table), whereas SPHK1, SPHK2, and SGPL1 were expressed similarly in most LN LEC subsets.

Previously, LN LECs have been implicated in the regulation of peripheral tolerance, expressing and presenting peripheral tissue self-antigens [5,6]. The lack of costimulatory molecules and their constitutive expression of T-cell–inhibitory molecules such as PD-L1 (CD274) leads to the elimination of potential autoreactive CD8^+^ T cells [6]. Subsequently, PD-L1 expression has been mapped to subcapsular and medullary LECs by flow cytometry and immunofluorescence staining in mouse LNs [19]. In agreement with this, we found PD-L1 transcript expression in fLECs as well as medullary/cortical LECs (S1 Table), suggesting that peripheral self-tolerance is primarily mediated by fLECs, cortical, and/or medullary LECs.

In a previous study, flow cytometry and laser-capture microdissection (LCM) were used to separate LECs in the SCS from other LN sinuses, followed by microarray analysis for transcriptional characterization [39]. However, it is unclear if LCM is sufficiently precise to separate individual sinus LECs from nearby cells such as sinus macrophages, and CD73 (5′-nucleotidase ecto [NT5E]), the marker used for FACS sorting of SCS versus other nodal LECs, was not differentially expressed in our single-cell RNA-sequencing data. Consequently, there was limited overlap between the transcriptional data presented in that study and our data set [39]. We observed a higher degree of overlap between our data and a more recently published data set of human LN LECs isolated from cervical and axillary LNs of tumor patients and subjected to single-cell RNA sequencing using the 10x Genomics platform [23]. Reminiscent of our findings, the authors described distinct subsets of fLECs, cLECs, and medullary LECs, which shared some up- and down-regulated genes with the corresponding mouse LN LEC subsets (Fig 9A–9C). This included, for instance, expression of CD36 in cLECs, suggesting that human cLECs may also have the capacity to scavenge modified LDLs. However, the majority of up- and down-regulated genes were different in those LEC subsets, most likely because of the differences of the investigated tissues (species, anatomical location, disease status) and the technologies used (tissue digestion and LEC isolation, sequencing platforms, etc.). Furthermore, Takeda and colleagues failed to detect a cluster of cortical, lymphocyte-egress–associated sinuses, possibly because of the 3′ transcriptomic profiling approach and the limited sequencing depth employed in their study [23]. On the other hand, we did not observe a specific medullary cLEC subpopulation as identified by Takeda and colleagues, which therefore appears to be human-specific.

Taken together, our data provide the first comprehensive transcriptional analysis of skin-draining LN LECs from naïve mice at the single-cell level, identifying LEC subsets, new marker genes, and subset-specific functions. It will be of great interest to investigate, in future studies, the specific changes in LEC subset composition and gene expression patterns in pathological conditions such as inflammation and cancer. Moreover, whole-transcriptome analysis of skin-draining LN LECs in comparison to those from, for instance, cervical and mandibular nodes that drain the brain [28] or mesenteric nodes might provide new insights into the cellular basis of internodal phenotypic and functional heterogeneity.

## Materials and methods

### Ethics statement

C57Bl/6N and congenic Prox1-GFP reporter mice [40] were bred in house in an SOPF-level facility. Ackr4-GFP mice [41] were kindly provided by Prof. Cornelia Halin (Institute of Pharmaceutical Sciences, ETH Zürich). All in vivo experiments were performed in agreement with national guidelines (Swiss Animal Experimentation Ordinance) and were approved by the responsible ethics committee (Kantonales Veterinäramt Zürich, license 5/18).

### Isolation of LN LECs

LECs were isolated from inguinal LNs of C57Bl/6N wild-type mice essentially as described before [42]. In brief, the LNs were dissected, and the capsule was broken using 23G injection needles. Subsequently, the tissue was digested in a solution containing 0.2 mg/ml Collagenase Type I (Worthington, Lakewood, NJ, USA), 0.8 mg/ml Dispase II, and 0.1 mg/ml DNAse I (both Roche, Basel, Switzerland) at 37°C. The samples were intermittently inverted or mixed by pipetting, and the entire digestion mix was renewed 2 times during the procedure. Once the tissue had completely dissolved, the cell suspension was washed with FACS buffer (1% FBS, 0.1 M EDTA, 0.02% NaN_3_), blocked with anti-CD16/32 (101302; Biolegend, San Jose, CA, USA), and labeled with anti-CD45.2-FITC (BD553772; BD Biosciences, San Diego, CA, USA), anti-CD31-APC (BD551262; BD Biosciences) and anti-Pdpn-PE (12-5381-82; Thermo Fisher Scientific, Waltham, MA, USA). Zombie-NIR (423106; Biolegend) was used for live/dead discrimination. Single, living CD45^-^ CD31^+^ Pdpn^+^ LECs were sorted directly into 384-well plates containing 0.8 μl of lysis buffer (0.1% Triton X-100, 2.5 mM dNTPs, 2.5 μM oligo-dT, 1 U/μl RNasin Plus RNase inhibitor; Promega, Madison, WI, USA) using a FACS Aria II instrument (BD Biosciences). Immediately after sorting, plates were centrifuged and stored at -80°C until further processing.

### Single-cell sequencing

Library preparation and sequencing were done at the Functional Genomics Center Zürich (FGCZ). In brief, the libraries were prepared using a miniaturized version of the Smart-seq2 protocol [43] with the help of a Mosquito HV pipetting robot (TTP Labtech, Melbourn, UK). Reverse transcription was performed in a final volume of 2 μl, followed by cDNA amplification in a final volume of 5 μl. The quality of the cDNAs was evaluated using a 2100 Bioanalyzer (Agilent, Santa Clara, CA, USA). 0.1 ng of cDNA from each cell on the plate was individually tagmented using the Nextera XT kit (Illumina, San Diego, CA, USA) in a final volume of 5 μl, followed by barcoding and library amplification in a final volume of 10 μl. The resulting 384 libraries were pooled, double-sided size selected (0.5× followed by 0.8× ratio using Ampure XP beads [Beckman Coulter, Brea, CA, USA]), and quantified using a 4200 TapeStation System (Agilent). The pool of libraries was sequenced in Illumina HiSeq2500 using single-read 125-bp chemistry with a depth of around 750,000 reads per cell (around 300 Mio reads per plate).

### Data processing, unsupervised clustering, and DE analyses

The Nextera adapter sequences and low-quality bases were removed using trimmomatic v0.33 [44]. Trimmed reads were aligned to the Ensembl mm10 mouse reference genome (release 92) using STAR v2.4.2a [45]. Gene expression quantification was computed with the “featureCounts” function in the Rsubread package v1.26.1 [46]. Quality filtering was performed with the scran package v1.4.5; cells with library size or feature size 2.5 median absolute deviations (MADs) away from the median or with mitochondrial contents 3 MADs above the median were dropped as outliers [47]. Genes expressed in at least 15% of cells were grouped in accordance with their count–depth relationship by SCnorm v0.99.7, which applied a quantile regression within each group to estimate scaling factors and normalize for sequencing depth [48]. Cells with detected CD45 expression were removed before downstream analyses.

To address the batch effects observed across different 384-well plates, we matched mutual nearest neighbors (MNNs) by applying the “fastMNN” function implemented in the batchelor package v1.0.1 [49]. Gene-specific variance was decomposed into biological and technical components. Variable features were defined as the genes displaying positive biological variances (FDR < 0.05, mean expression level between 0.25 and 4). The variable features were then subjected to merged principal component analysis (PCA) across all batches. Unsupervised clustering was performed on the corrected low-dimensional coordinates (PC 1–10) using the Seurat package v2.3.4 [50] and visualized with Uniform Manifold Approximation and Projection (UMAP) [51]. Differentially expressed genes in each cluster compared to all other clusters were identified by the “FindMarkers” function (min.pct = 0.20, logfc.threshold = 0.6, p_val_adj < 0.01) using the MAST test [52]. The expression patterns of selected markers were plotted by the “FeaturePlot” function using the corrected expression values. The entire gene expression data are available at ArrayExpress with accession number E-MTAB-8414.

### Analysis and comparison of previously published human LN LEC data

Raw data were downloaded from GSE124494 and reanalyzed with Seurat. Quality control was performed as previously described [23]. Six human LN data sets were aligned using Canonical Correlation Analysis (CCA), with highly variable genes identified in at least 2 data sets. Clusters were defined using the aligned canonical correlation vectors (CC) 1–30 and resolution 0.5. Only the LEC population was subsetted for downstream analysis. Differentially expressed genes among clusters were identified with “FindAllMarkers” (min.pct = 0.25, logfc.threshold = 0.25, and p_val_adj < 0.05). Genes up-regulated in LEC I, II, and VI were compared to genes up-regulated in our cLEC, fLEC, and medullary LEC clusters, respectively. Orthologous genes were converted using the biomaRt package [53].

### Immunofluorescence staining

Inguinal LNs of C57Bl/6N wild-type mice were dissected, embedded in OCT compound, and frozen in liquid nitrogen. In the case of Ackr4-GFP reporter mice, inguinal, mandibular, iliac, and mesenteric LNs were dissected, fixed with 2% paraformaldehyde for 2 h at room temperature, treated with 1 M sucrose overnight, embedded in OCT compound, and frozen in liquid nitrogen. Seven-μm sections were cut with a cryostat, fixed in ice-cold acetone and 80% methanol, rehydrated in PBS, and subsequently blocked in PBS + 0.2% BSA, 5% donkey serum, 0.3% Triton-X100, and 0.05% NaN_3_ (blocking solution). Primary antibodies included goat anti-LYVE1 (AF2125; R&D, Minneapolis, MN, USA), rabbit anti-LYVE1 (11–034; AngioBio, San Diego, CA, USA), rat anti-LYVE1 (NBP1-43411; Novus Biologicals, Centennial, CO, USA), goat anti-prox1 (AF2727; R&D), rabbit anti-CD3 (NB600-1441; Novus Biologicals), rat anti-IgD (1120–01; SouthernBiotech, Birmingham, AL, USA), rat anti-CD4 (BD553647; BD Biosciences), rat anti-F4/80 (MCA487R; Bio-Rad, Hercules, CA, USA), rat anti-CD169 (MCA884; Bio-Rad), rat anti-ITGA2B (BD553847; BD Bioscience), rat anti-CD44 (103002; Biolegend), rabbit anti-ANXA2 (ab178677; Abcam, Cambridge, UK), rabbit anti-FABP4 (15872-1-AP; ProteinTech, Rosemont, IL, USA), goat anti-CD36 (AF2519, R&D), goat anti-IL33 (AF3626; R&D), goat anti-MRC1 (AF2535; R&D), rat anti-MARCO (GTX39744; Genetex, Irvine, CA, USA), rat anti-MADCAM1 (BD553805; BD Bioscience), rabbit anti-BGN (HPA003157; Sigma-Aldrich, St. Louis, MO, USA), and goat anti-FLRT2 (AF2877; R&D). They were suspended in blocking solution and incubated at 4°C overnight, followed by incubation with Alexa488-, Alexa594-, or Alexa647-conjugated secondary antibodies (donkey anti-rat, donkey anti-rabbit, donkey anti-goat; Thermo Fisher Scientific) together with Hoechst33342 (Sigma-Aldrich) for nuclear counterstaining. Images were captured with an LSM 780 upright confocal microscope (Zeiss, Jena, Germany) and analyzed with Fiji [54].

### RNA fluorescence in situ hybridization

RNA fluorescence in situ hybridization (FISH) was performed using the RNAscope Multiplex Fluorescent Reagent Kit v2 (ACD, Newark, CA, USA) according to the manufacturer’s instructions. Inguinal LNs were fixed with 10% neutral-buffered paraformaldehyde and embedded in paraffin for sectioning (7 μm). Antigen retrieval was performed with Target Retrieval Reagent for 15 min after deparaffinization. Slides were treated with Protease Plus for 30 min, incubated with probes in the ACD HybEZ hybridization system, and stained with Opal 570. The following RNAscope probes were used: Ackr3 (C1), Btln9 (C1), Coch (C3), Glycam1 (C1), Itih5 (C1), Kcnj8 (C1), and Ptx3 (C1). Slides were stained with primary antibodies as described above, followed by incubation with Alexa488- and/or Alexa647-conjugated secondary antibodies together with Hoechst33342 for nuclear counterstaining, and mounted with ProLong Gold Antifade Mounting medium (Thermo Fisher Scientific). Images were captured with an LSM 780 upright confocal microscope and analyzed with Fiji.

### Flow cytometry analysis

Inguinal LNs from C57Bl/6N wild-type and Ackr4-GFP were processed, washed, and Fc-blocked as described above (Isolation of LN LECs). Subsequently, cell suspensions were stained with anti-CD45-PacificBlue (103126; Biolegend), anti-Pdpn-PE, anti-CD31-PerCp/Cy5.5 (102522; Biolegend), anti-ITGA2B-BV421 (133911; Biolegend), anti-MRC1-APC (141708; Biolegend), anti-CD44-BV650 (103049; Biolegend), and Zombie-NIR and analyzed on a LSRFortessa flow cytometer (BD Biosciences).

### Light sheet microscopy

Inguinal LNs derived from Prox1-GFP reporter mice were fixed with 4% paraformaldehyde for 2 h at room temperature, permeabilized in 0.5% Triton X-100 in PBS for 2 days, and stained with chicken anti-GFP (GFP1010; Aves Labs, Davis, CA, USA) and rabbit anti-ANXA2 for 7 days, followed by incubation with Alexa488- and Alexa594-conjugated secondary antibodies for 7 days. Optical clearing was performed as described previously [13]. In brief, stained LNs were embedded in 1% ultrapure LMP Agarose (Thermo Fisher Scientific) on ice; dehydrated in a series of 50%, 70%, 95%, and 100% methanol; and precleared with 50% BABB (benzyl alcohol/benzyl benzoate 1:2) in methanol, followed by clearing in 100% BABB overnight. Wholemount images were captured with an UltraMicroscope I (LaVision Biotec, Bielefeld, Germany) and analyzed with Fiji.

### In vivo LDL tracing assay

10 μg of Dil-labeled human acetylated LDL (Kalen Biomedical, Germantown, MD, USA) or 10 μg of Dil-labeled human oxidized LDL (Thermo Fisher Scientific) was intradermally injected unilaterally close to the base of the tail of Ackr4-GFP reporter mice under isoflurane anesthesia. An equal volume of PBS was injected on the opposite side as a control. Draining inguinal LNs were collected 1 h later, fixed with 2% paraformaldehyde for 2 h at room temperature, embedded in OCT compound, and frozen in liquid nitrogen. Immunofluorescence staining using a goat anti-LYVE1 antibody (R&D) followed by incubation with an Alexa647-conjugated secondary antibody together with Hoechst33342 (Sigma-Aldrich) was performed on 7-μm sections without acetone fixation. Images covering the whole SCS and SCS/CS regions were captured with an LSM 780 upright confocal microscope. The intensity of LDL staining in the ACKR4^+^ area and the ACKR4^-^ LYVE1^+^ area was measured with Fiji. For quantification, the average signal intensity of all images representing each individual mouse was normalized (ACKR4^-^ LYVE1^+^ area = 1).

### Adoptive lymphocyte transfer and tracing

Splenocytes were collected from naïve C57Bl/6N wild-type mice after lysis of red blood cells with PharmLyse buffer (BD Bioscience) and were labeled with 5 mM of CFSE (Sigma-Aldrich) in PBS for 15 min at 37°C. Then, 2 × 10^6^ labeled splenocytes were infused into the tail vein of sex-matched recipient mice. Inguinal LNs were collected 30 min later, fixed with 10% paraformaldehyde overnight at room temperature, and embedded in paraffin. Seven-μm and 40-μm sections were deparaffinized, followed by antigen retrieval (10 mM citrate buffer [pH 6.0]) and immunofluorescence staining using goat anti-LYVE1 (R&D) and rabbit anti-ANXA2 (Abcam) antibodies and donkey anti-goat and anti-rabbit Alexa594- and Alexa647-conjugated secondary antibodies. Images covering all cortical and medullary regions were captured with an LSM 780 upright confocal microscope and analyzed with Fiji. The number of CFSE-labeled cells in ANXA2^+^ sinuses and in ANXA2^-^ sinuses were counted manually using 7-μm sections. Maximum intensity projections of confocal z-stack images were prepared using 40-μm sections of the same samples.

### Statistical analysis

Statistical analysis was performed using GraphPad Prism (GraphPad Software, San Diego, CA). Student *t* test was used for comparisons of 2 groups. A *p*-value < 0.05 was considered statistically significant. ScRNA-seq data analyses and graphical interpretation were performed using R v3.6.1.

## Supporting information

S1 Table Gene signatures of LN LEC subtypes. List of genes differentially expressed (log_2_FC ≥ 0.6; FDR < 0.01) among 4 clusters of LECs. FDR, false discovery rate; LEC, lymphatic endothelial cell; LN, lymph node.
(XLSX)

S2 Table GO analysis of LN LEC subtypes. GO analysis of biological process (GO_BP) terms using differentially expressed genes among the 4 LN LEC clusters. Only the top 10 most significantly enriched terms are shown for each of the clusters. GO, gene ontology; LEC, lymphatic endothelial cell; LN, lymph node.
(XLSX)

S3 Table Gene-level comparison between mouse and human LN LEC subtypes. Gene-level comparison between cLECs, fLECs, and medullary LECs in mice and human (Takeda and colleagues [23]). Both up- and down-regulated genes in each of the subpopulations (compared to all other LECs) were compared. Highlighted genes are conserved between the data sets. cLEC, ceiling LEC; fLEC, floor-lining LEC; LEC, lymphatic endothelial cell; LN, lymph node.
(XLSX)

S1 Fig Additional characterization of fLECs. (A) Immunofluorescence staining for LYVE1 (green) and MADCAM1 (red), showing specific MADCAM1 staining in the floor of the subcapsular sinus. (B) Immunofluorescence staining for LYVE1 (green) and ITGA2B (red). LYVE1 and ITGA2B were clearly detectable in fLECs and cortical LECs in both the SCS and the IF-SCS regions. (C, D) Immunofluorescence staining for LYVE1 (green) in Ackr4-GFP reporter mice. ACKR4+ cLECs (white) were detected in both the SCS region and the IF-SCS region. ACKR4, atypical chemokine receptor 4; cLEC, ceiling LEC; fLEC, floor-lining LEC; GFP, green fluorescent protein; IF, interfollicular; ITGA2B, integrin subunit alpha 2b; LEC, lymphatic endothelial cell; LYVE1, lymphatic vessel endothelial hyaluronan receptor 1; MADCAM1, mucosal vascular addressin cell adhesion molecule 1; SCS, subcapsular sinus.
(PDF)

S2 Fig Additional characterization of cLECs. (A) Light sheet fluorescence microscopy image of an optically cleared inguinal LN derived from a Prox1-GFP reporter mouse. Immunofluorescence staining for ANXA2 (red) revealed that afferent lymphatic collectors express ANXA2 (white arrowhead). (B, C) Expression of new cLEC/cluster 2 marker genes BGN (B) and FLRT2 (C) by RNA sequencing (left panels) and immunofluorescence staining (right panels) in Ackr4-GFP reporter mice. GFP (white) and immunofluorescence costaining for LYVE1 (green) served as markers for cLECs and fLECs, respectively. ACKR4, atypical chemokine receptor 4; ANXA2, annexin A2; BGN, biglycan; cLEC, ceiling LEC; fLEC, floor-lining LEC; FLRT2, fibronectin leucine-rich transmembrane protein 2; GFP, green fluorescent protein; LEC, lymphatic endothelial cell; LN, lymph node; LYVE1, lymphatic vessel endothelial hyaluronan receptor 1; Prox1, prospero homeobox 1.
(PDF)

S3 Fig LN LEC subtype marker expression in other LNs. (A–D) Immunofluorescence images of mandibular, iliac, and mesenteric LN sections derived from Ackr4-GFP reporter mice, stained for LYVE1 (green), CD44 (red) (A), ANXA2 (red) (B), CD36 (red) (C), or MRC1 (red) (D). GFP fluorescence is shown in white. ACKR4, atypical chemokine receptor 4; ANXA2, annexin A2; CD, cluster of differentiation; GFP, green fluorescent protein; LEC, lymphatic endothelial cell; LN, lymph node; LYVE1, lymphatic vessel endothelial hyaluronan receptor 1; MRC1, mannose receptor C-type 1.
(PDF)

S4 Fig Identification of LN LEC subtypes by flow cytometry. (A) Gating strategy to identify the major LEC subsets (cLECs, fLECs, medullary LECs) in inguinal LNs from Ackr4-GFP mice by flow cytometry. Within LN stromal cells (pregated as CD45−, Zombie-NIR− singlets), LECs were identified as PDPN+ CD31+ cells. cLECs were identified by GFP expression. Among the remaining cells, medullary LECs expressed MRC1 and were predominantly ITGA2B+, whereas fLECs were MRC1− but expressed relatively high levels of CD44 and ITGA2B. (B) Staining controls for GFP (using wild-type C57Bl/6N mice), MRC1, ITGA2B, and CD44. (C) Intensity histograms for GFP, MRC1, ITGA2B, and CD44 in cLECs (green curve), fLECs (orange curve), and medullary LECs (blue curve) identified as shown in panel A. ACKR4, atypical chemokine receptor 4; CD, cluster of differentiation; cLEC, ceiling LEC; fLEC, floor-lining LEC; GFP, green fluorescent protein; ITGA2B, integrin subunit alpha 2b; LEC, lymphatic endothelial cell; LN, lymph node; MRC1, mannose receptor C-type 1; NIR, near infrared; PDPN, podoplanin.
(PDF)

S5 Fig Additional characterization of cluster 4 LECs. (A–D) Representative images of cluster 4 sinuses stained for LYVE1 (green) and ANXA2 (red). The location relative to major immune cell populations is shown by staining for IgD (A), CD4 (B), F4/80 (C), and CD169 (D). (D–G) Immunofluorescence staining for LYVE1 (green), ANXA2 (red), and PROX1 (A), MRC1 (B), and MARCO (C) (white). White arrowheads indicate LYVE1+/ANXA2+ cells. ANXA2, annexin A2; CD, cluster of differentation; IgD, immunoglobulin D; LEC, lymphatic endothelial cell; LYVE1, lymphatic vessel endothelial hyaluronan receptor 1; MARCO, macrophage receptor with collagenous structure; MRC1, mannose receptor C-type 1; PROX1, prospero homeobox 1.
(PDF)

S1 Data Quantitative data related to Figs 5, 8 and 9. Supplementary data file with raw quantification results related to Figs 5, 8 and 9.
(XLSX)

## Abbreviations

ACKR4: atypical chemokine receptor 4
ANXA2: annexin A2
ApoB: apolipoprotein B
BABB: benzyl alcohol/benzyl benzoate
BGN: biglycan
Btnl9: butyrophilin like 9
CCA: Canonical Correlation Analysis
CD: cluster of differentiation
CFSE: carboxy-fluorescein diacetate succinimidyl ester
cLEC: ceiling LEC
Coch: cochlin
DE: differential expression
FABP4: fatty acid binding protein 4
FACS: fluorescence-activated cell sorting
Fcgr2b: Fc fragment of IgG receptor IIb
FDR: false discovery rate
FGCZ: Functional Genomics Center Zürich
FISH: fluorescence in situ hybridization
fLEC: floor-lining LEC
Flt4: Fms related receptor tyrosine kinase 4
FLRT2: fibronectin leucine-rich transmembrane protein 2
FRC: fibroblastic reticular cell
GFP: green fluorescent protein
Glycam1: glycosylation-dependent cell adhesion molecule 1
GO: gene ontology
HEV: high endothelial venule
IF: interfollicular
IL33: interleukin 33
ITGA2B: integrin subunit alpha 2b
Itih5: inter-alpha-trypsin inhibitor heavy chain 5
Kcnj8: potassium inwardly rectifying channel subfamily J member 8
LCM: laser-capture microdissection
LDL: low-density lipoprotein
LEC: lymphatic endothelial cell
LN: lymph node
LYVE1: lymphatic vessel endothelial hyaluronan receptor 1
MAD: median absolute deviation
MADCAM1: mucosal vascular addressin cell adhesion molecule 1
MARCO: macrophage receptor with collagenous structure
MNN: mutual nearest neighbor
MRC1: mannose receptor C-type 1
MS: medullary sinus
Msr1: macrophage scavenger receptor 1
NT5E: 5′-nucleotidase ecto
PCA: principal component analysis
PDGF: platelet-derived growth factor
Pdpn: podoplanin
PD-L1: programmed death ligand 1
Pecam1: platelet and endothelial cell adhesion molecule 1
Prox1: prospero homeobox 1
Ptx3: pentraxin 3
SCS: subcapsular sinus
SGPL1: S1P lyase
SPHK: sphingosine kinase
SPNS2: sphingolipid transporter 2
UMAP: Uniform Manifold Approximation and Projection
VEGF: vascular endothelial growth factor
