# Supplementary figures and images for: Single-cell mapping reveals new markers and functions of lymphatic endothelial cells in lymph nodes
Source: PLoS Biol. 2020 Apr 6;18(4):e3000704. doi: 10.1371/journal.pbio.3000704 (PMC7162550; doi:10.1371/journal.pbio.3000704)

Figure S1

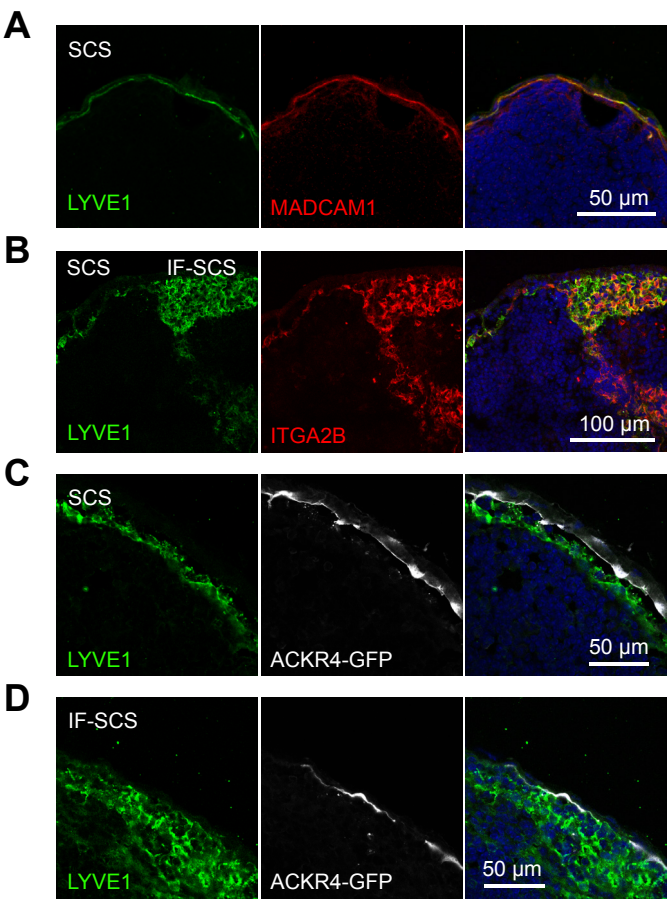

Supplement: S1 Fig — (A) Immunofluorescence staining for LYVE1 (green) and MADCAM1 (red), showing specific MADCAM1 staining in the floor of the subcapsular sinus. (B) Immunofluorescence staining for LYVE1 (green) and ITGA2B (red). LYVE1 and ITGA2B were clearly detectable in fLECs and cortical LECs in both the SCS and the IF-SCS regions. (C, D) Immunofluorescence staining for LYVE1 (green) in Ackr4-GFP reporter mice. ACKR4+ cLECs (white) were detected in both the SCS region and the IF-SCS region. ACKR4, atypical chemokine receptor 4; cLEC, ceiling LEC; fLEC, floor-lining LEC; GFP, green fluorescent protein; IF, interfollicular; ITGA2B, integrin subunit alpha 2b; LEC, lymphatic endothelial cell; LYVE1, lymphatic vessel endothelial hyaluronan receptor 1; MADCAM1, mucosal vascular addressin cell adhesion molecule 1; SCS, subcapsular sinus. (PDF) [file pbio.3000704.s004.pdf]

## Figure S2

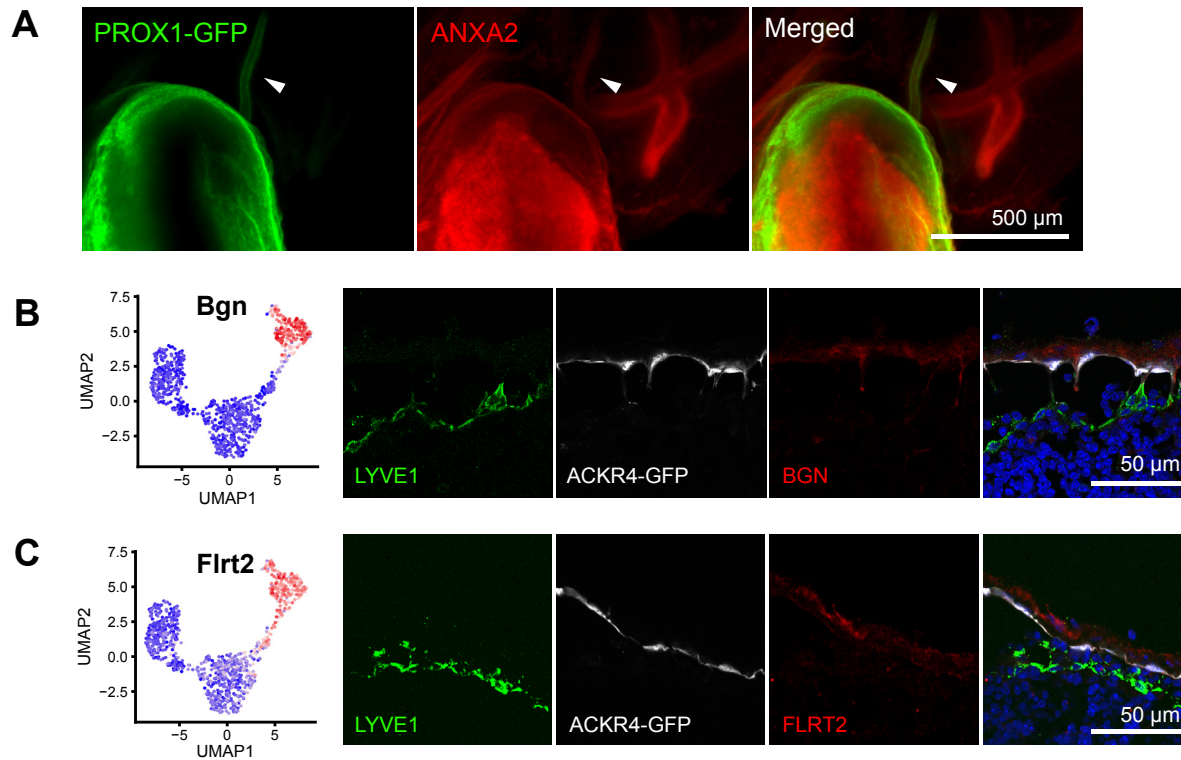

Supplement: S2 Fig — (A) Light sheet fluorescence microscopy image of an optically cleared inguinal LN derived from a Prox1-GFP reporter mouse. Immunofluorescence staining for ANXA2 (red) revealed that afferent lymphatic collectors express ANXA2 (white arrowhead). (B, C) Expression of new cLEC/cluster 2 marker genes BGN (B) and FLRT2 (C) by RNA sequencing (left panels) and immunofluorescence staining (right panels) in Ackr4-GFP reporter mice. GFP (white) and immunofluorescence costaining for LYVE1 (green) served as markers for cLECs and fLECs, respectively. ACKR4, atypical chemokine receptor 4; ANXA2, annexin A2; BGN, biglycan; cLEC, ceiling LEC; fLEC, floor-lining LEC; FLRT2, fibronectin leucine-rich transmembrane protein 2; GFP, green fluorescent protein; LEC, lymphatic endothelial cell; LN, lymph node; LYVE1, lymphatic vessel endothelial hyaluronan receptor 1; Prox1, prospero homeobox 1. (PDF) [file pbio.3000704.s005.pdf]

Figure S3

A

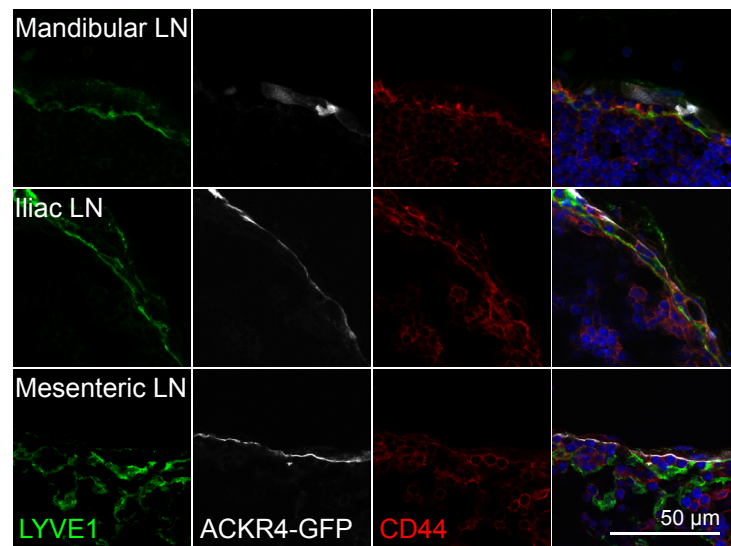

B

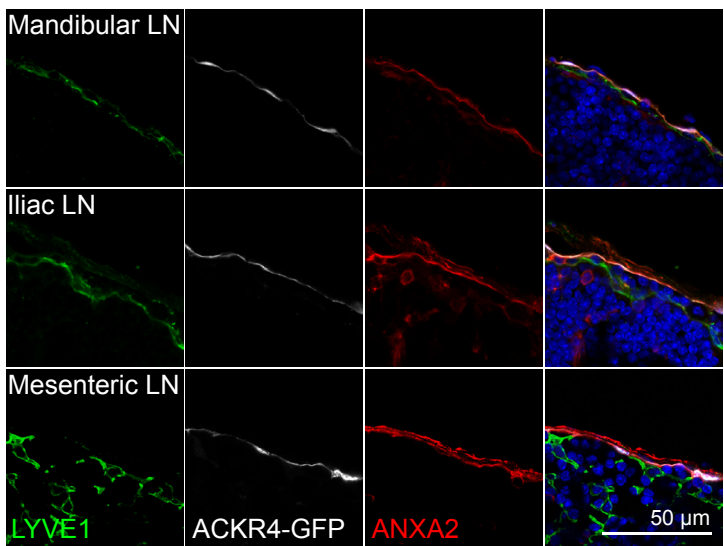

C

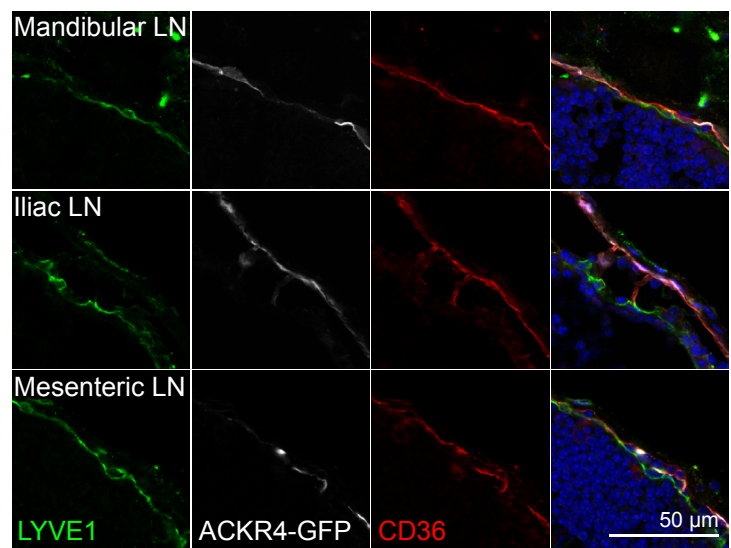

D

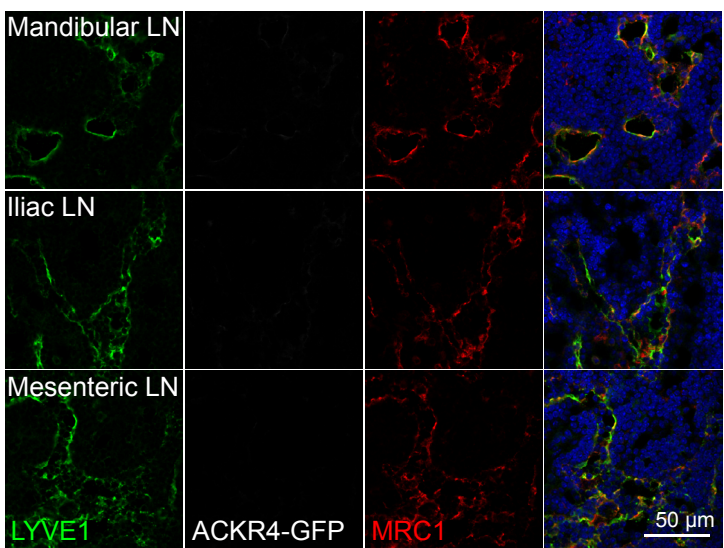

Supplement: S3 Fig — (A–D) Immunofluorescence images of mandibular, iliac, and mesenteric LN sections derived from Ackr4-GFP reporter mice, stained for LYVE1 (green), CD44 (red) (A), ANXA2 (red) (B), CD36 (red) (C), or MRC1 (red) (D). GFP fluorescence is shown in white. ACKR4, atypical chemokine receptor 4; ANXA2, annexin A2; CD, cluster of differentiation; GFP, green fluorescent protein; LEC, lymphatic endothelial cell; LN, lymph node; LYVE1, lymphatic vessel endothelial hyaluronan receptor 1; MRC1, mannose receptor C-type 1. (PDF) [file pbio.3000704.s006.pdf]

Figure S4

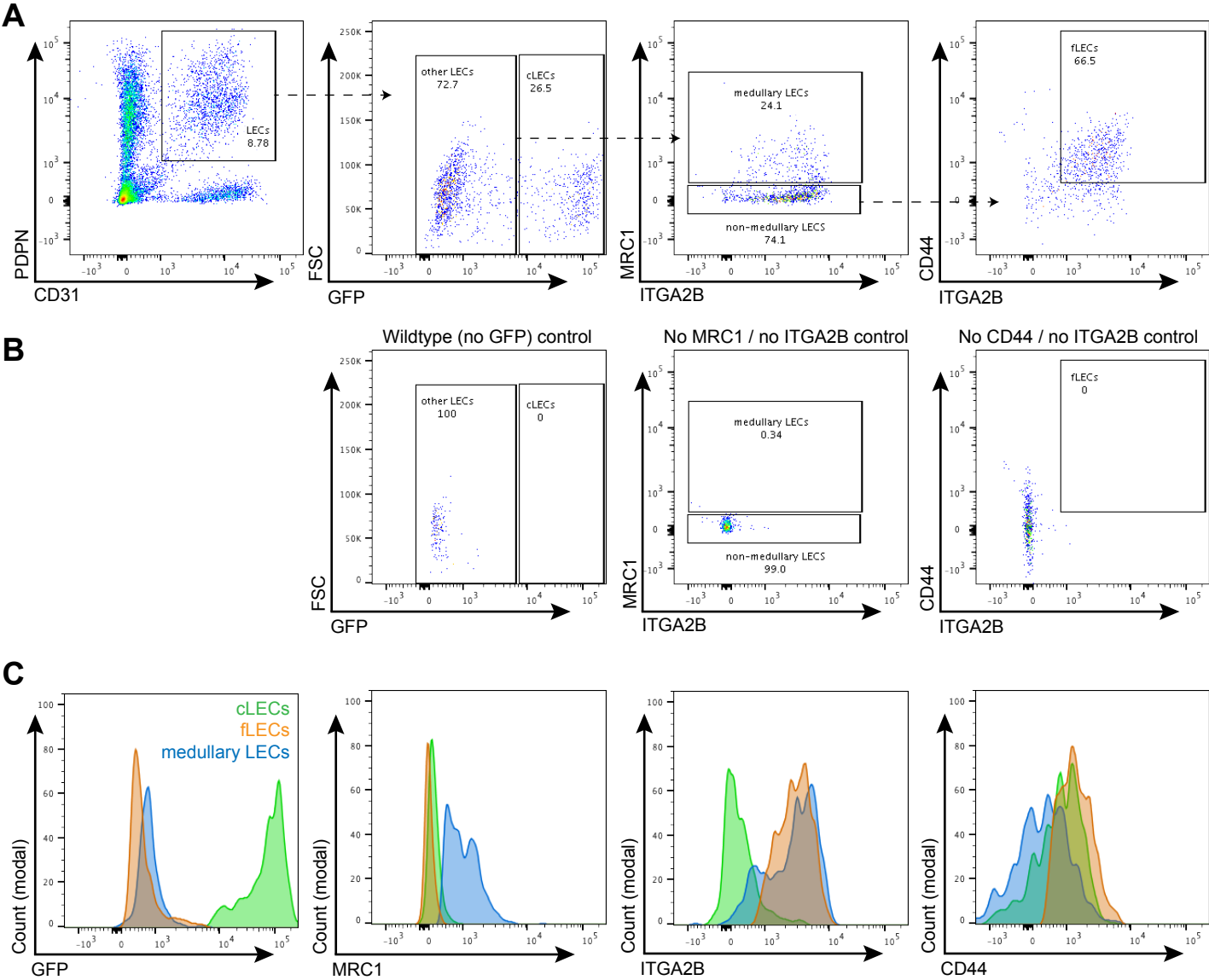

Supplement: S4 Fig — (A) Gating strategy to identify the major LEC subsets (cLECs, fLECs, medullary LECs) in inguinal LNs from Ackr4-GFP mice by flow cytometry. Within LN stromal cells (pregated as CD45−, Zombie-NIR− singlets), LECs were identified as PDPN+ CD31+ cells. cLECs were identified by GFP expression. Among the remaining cells, medullary LECs expressed MRC1 and were predominantly ITGA2B+, whereas fLECs were MRC1− but expressed relatively high levels of CD44 and ITGA2B. (B) Staining controls for GFP (using wild-type C57Bl/6N mice), MRC1, ITGA2B, and CD44. (C) Intensity histograms for GFP, MRC1, ITGA2B, and CD44 in cLECs (green curve), fLECs (orange curve), and medullary LECs (blue curve) identified as shown in panel A. ACKR4, atypical chemokine receptor 4; CD, cluster of differentiation; cLEC, ceiling LEC; fLEC, floor-lining LEC; GFP, green fluorescent protein; ITGA2B, integrin subunit alpha 2b; LEC, lymphatic endothelial cell; LN, lymph node; MRC1, mannose receptor C-type 1; NIR, near infrared; PDPN, podoplanin. (PDF) [file pbio.3000704.s007.pdf]

Figure S5

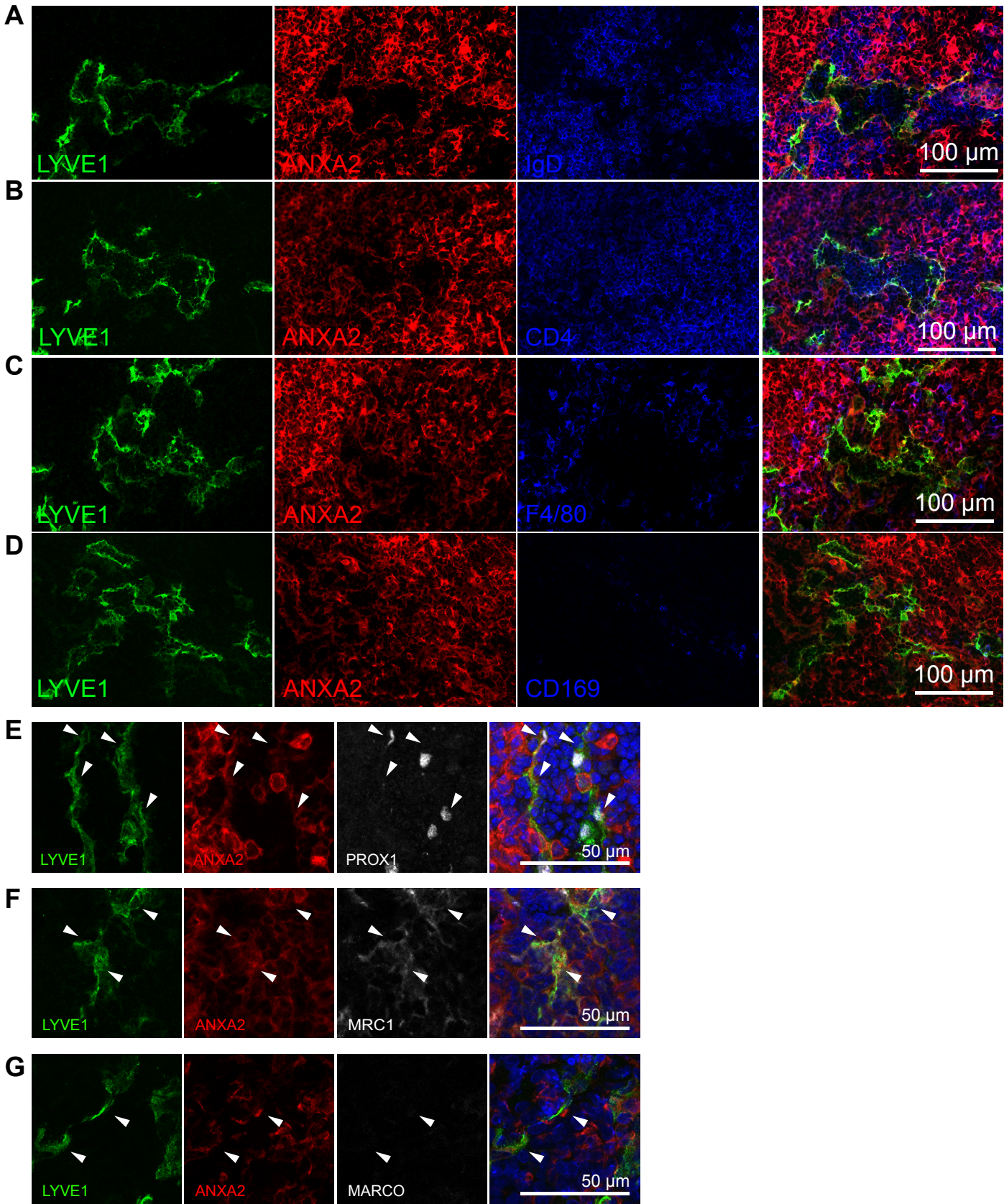

Supplement: S5 Fig — (A–D) Representative images of cluster 4 sinuses stained for LYVE1 (green) and ANXA2 (red). The location relative to major immune cell populations is shown by staining for IgD (A), CD4 (B), F4/80 (C), and CD169 (D). (D–G) Immunofluorescence staining for LYVE1 (green), ANXA2 (red), and PROX1 (A), MRC1 (B), and MARCO (C) (white). White arrowheads indicate LYVE1+/ANXA2+ cells. ANXA2, annexin A2; CD, cluster of differentation; IgD, immunoglobulin D; LEC, lymphatic endothelial cell; LYVE1, lymphatic vessel endothelial hyaluronan receptor 1; MARCO, macrophage receptor with collagenous structure; MRC1, mannose receptor C-type 1; PROX1, prospero homeobox 1. (PDF) [file pbio.3000704.s008.pdf]
